# Hijacking competitor-derived signals: RcsB/C drives *Lysobacter enzymogenes* to exploit farnesol for enhanced antifungal capacity

**DOI:** 10.1128/aem.00304-26

**Published:** 2026-04-03

**Authors:** Fang Nan, Huihui Song, Min Sun, Lei Cui, Zeran Bian, Jie Yin, Zipeng Lin, Yan Wang

**Affiliations:** 1 Marine Microbial Resource Center, MOE Key Laboratory of Evolution and Marine Biodiversity, College of Marine Life Sciences, Ocean University of China 506915 , Qingdao, China; 2 Institute of Evolution and Marine Biodiversity, Ocean University of China 535359 , Qingdao, China; 3 Laboratory for Marine Ecology, Qingdao Marine Science and Technology Center & Laoshan Laboratory 474988, Qingdao, China; The University of Arizona, Tucson, Arizona, USA

**Keywords:** bacterial-fungal interaction, cross-kingdom communication, farnesol, *Lysobacter enzymogenes*, two-component system, HSAF

## Abstract

Microbial antagonism is a fundamental ecological process that shapes community composition and maintains ecosystem balance. However, the molecular signals, such as cross-kingdom interactions between bacteria and fungi in natural environments, remain largely unexplored. *Lysobacter enzymogenes* is a widespread predatory bacterium that produces the antifungal secondary metabolite heat-stable antifungal factor (HSAF), which enables interactions with diverse fungi. Here, we investigated the interkingdom interactions between *L. enzymogenes* and the environmental fungus *Candida krusei*, both of which are widely distributed, to elucidate the bacterial-fungal communication. We found that the antagonistic effect of bacteria-fungi was significant under both contact and non-contact co-culture conditions, indicating the involvement of diffusible metabolites. Given that farnesol is a common quorum-sensing (QS) molecule in *Candida*, metabolite profiling combined with exogenous addition and biosynthesis inhibition experiments demonstrated that farnesol functions as a cross-kingdom signal regulating HSAF production and bacterial antagonism. Further mechanistic analysis of the intrinsic mechanism revealed that the two-component system (TCS) RcsB/C in *L. enzymogenes* can sense farnesol and activate the production of HSAF through the MarR family regulators, mediating the antagonistic pathway. In addition, our work identified the key amino acid residues in RcsC of *L. enzymogenes* responsible for recognizing farnesol. In summary, we report the bacterial TCS involved in farnesol sensing and reveal a novel bacterial-fungal antagonistic mechanism, in which *L. enzymogenes* “hijacks” a fungal QS molecule to enhance its antifungal capacity, uncovering a previously unrecognized strategy of cross-kingdom communication.

**IMPORTANCE:**

Bacteria and fungi frequently interact in shared habitats, yet the chemical cues that shape these cross-kingdom relationships remain poorly defined. Farnesol is a well-known quorum-sensing molecule in *Candida*, but its ecological roles beyond fungal communication are unclear. Here, we show that *Lysobacter enzymogenes* directly senses fungal-derived farnesol through the RcsB/C two-component system, which activates the downstream regulator MarR-2 and induces the production of the antifungal metabolite heat-stable antifungal factor (HSAF). This signal hijacking strategy allows *L. enzymogenes* to convert a fungal communication molecule into a cue that strengthens its antagonistic capacity. We further identify key amino acid residues in RcsC responsible for farnesol recognition, revealing the bacterial two-component system (TCS) known to detect this molecule. These findings expand the functional scope of fungal quorum-sensing signals, uncover a previously unrecognized mechanism of interkingdom antagonism, and provide insights with potential applications in microbiome-based biocontrol.

## INTRODUCTION

Microbial antagonism is a fundamental ecological process that profoundly shapes microbial community composition, interspecies competition, and ecosystem stability (1, 2). Bacteria and fungi often coexist in complex habitats such as soil, plant rhizosphere, and host-associated environments, where they compete for limited nutrients and space (3–6). In such environments, antagonistic interactions are one of the central factors determining microbial fitness and shaping population structures (3, 7, 8). Such interactions are mediated by diverse strategies (5, 8–10), including the secretion of antibiotics or extracellular enzymes, as well as direct physical contact. Although these mechanisms have been widely investigated, the molecular cues that regulate such cross-kingdom antagonism remain poorly understood (11, 12). Dissecting these signaling processes is key to elucidating how microbes communicate, adapt, and survive within competitive ecological niches. An increasing number of studies have emphasized the importance of small signaling molecules in mediating interkingdom interactions (13–15). In fungi, quorum-sensing (QS) molecules (16–19), such as farnesol and tyrosol, regulate morphogenesis, biofilm formation, and virulence. In bacteria, quorum sensing (20, 21) and two-component regulatory systems (21, 22) play critical roles in coordinating collective behaviors and secondary metabolism. Despite increasing evidence of bacterial-fungal chemical dialogs, most studies have focused on fungal responses to bacterial metabolites. To date, it remains largely unknown whether specifically bacterial predators can respond to quorum-sensing signals from fungal antagonists during antagonism, and whether they exploit such signals to enhance their own antagonistic activity.

*Lysobacter enzymogenes* is a widely distributed, environmentally predatory Gram-negative bacterium that has been studied for its remarkable antifungal capacity (23, 24). It produces the heat-stable antifungal factor (HSAF), a polycyclic tetramate macrolactam that disrupts fungal sphingolipid biosynthesis and inhibits fungal development (25–27). HSAF biosynthesis is tightly controlled by multilayered regulatory networks (28–30), including global regulators and two-component systems (TCSs), highlighting the role as an important ecological strategy for microbial competition. Previous studies have shown that *L. enzymogenes* can effectively antagonize a wide range of fungi in soil and plant-associated environments (5, 23, 31). Furthermore, we first discovered that the TCS RcsB/C in *L. enzymogenes* can sense fungal farnesol and identified the key residues responsible for this recognition. RcsB/C significantly enhances HSAF biosynthesis through the downstream MarR family transcriptional regulator. This work reveals a novel regulatory mechanism in which *L. enzymogenes* exploits fungal QS signals to enhance its antifungal capability, thereby strengthening its competitive advantage. By effectively “hijacking” competitor-derived signals and turning the opponent’s signals against them, this strategy provides fresh theoretical insights into the molecular mechanisms and regulatory pathways driving bacterial-fungal antagonism.

## RESULTS

### Verification of bacterial-fungal antagonism and identification of farnesol as a chemical signal

**Fig 1:**
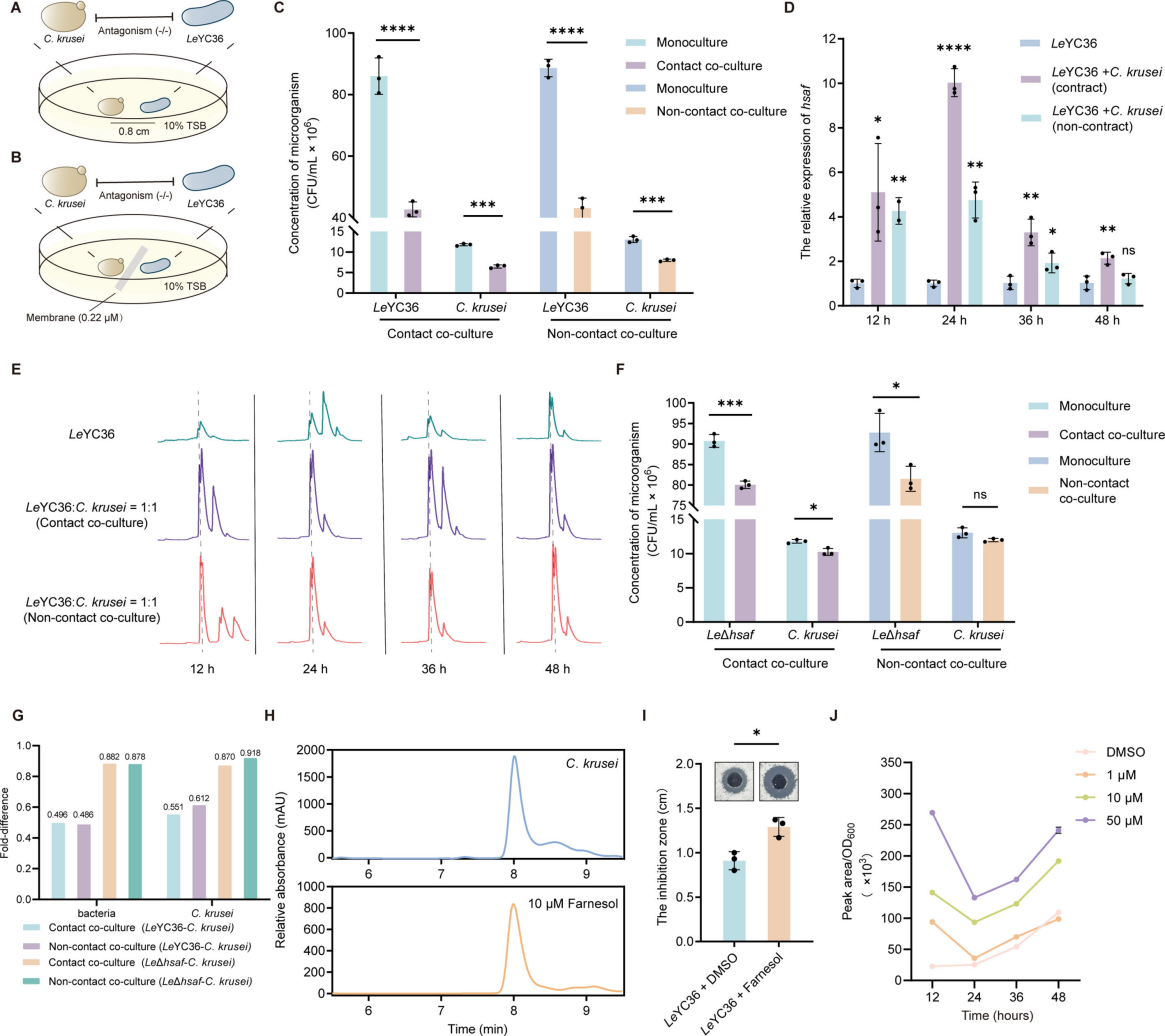
Farnesol-mediated regulation of HSAF synthesis during *Lysobacter enzymogenes* YC36 and *Candida krusei* antagonistic interactions. (**A, B**) Schematic diagrams of the contact co-culture system (**A**) and the non-contact co-culture system (**B**) of *Le*YC36 with *C. krusei*. (**C**) Biomass changes of *Le*YC36 and *C. krusei* in contact and non-contact co-culture systems were determined by CFU counting. (**D**) Transcriptional levels of the *hsaf* gene under co-culture conditions by RT-qPCR. (**E**) HSAF biosynthesis levels during co-culture were determined by HPLC. (**F**) Biomass changes of the *hsaf* deletion mutant (*Le*Δ*hsaf*) and *C. krusei* in contact and non-contact systems. (**G**) A side-by-side comparison of the fold difference of CFU in mono- and co-culture of wild-type *Le*YC36, *Le*Δ*hsaf,* and *C. krusei*. Fold difference was calculated as the ratio of CFU mean in co-culture to that in mono-culture. (**H**) HPLC detection of farnesol secreted extracellularly by *C. krusei*. (**I**) The enhancement of the inhibitory effect of *Le*YC36 on *C. krusei* via exogenous farnesol addition was evaluated by the agar diffusion assay. (**J**) Effects of different concentrations of farnesol on HSAF production in *Le*YC36 by HPLC. For panels A and B, the thickness of the lines represents the strength of the antagonistic relationship. (−/−) indicates that the biomass of both organisms decreases, showing an antagonistic trend. For panels C, D, F, I, and J, error bars indicate the standard deviation (SD) of the three replicates. Data presented as mean ± SD. For significance information, ns, not significant, *P value < 0.05, **P value < 0.01, ***P value < 0.001, ****P value < 0.0001.

The underlying mechanisms of cross-kingdom interactions between bacteria and fungi, and the signaling cues that mediate these interactions, remain largely unexplored. To address this question, we investigated the interaction between *Lysobacter enzymogenes* YC36 (*Le*YC36) and the fungus *Candida krusei*. Both contact and non-contact co-culture assays revealed a significant reduction in the biomass of both organisms (Fig. 1A through C), demonstrating a strong antagonistic interaction between them. Based on these results, we further explored the molecular mechanisms underlying this antagonism. Considering that HSAF is the major antifungal compound produced by *L. enzymogenes* (32)*,* we next examined whether it mediates this antagonism and whether its biosynthesis is affected under coculture conditions. Indeed, both HSAF biosynthetic gene expression and metabolite accumulation were markedly upregulated in both contact and non-contact systems (Fig. 1D and E), suggesting that HSAF contributes to the antagonistic interaction between *Le*YC36 and *C. krusei*. Furthermore, deletion of the HSAF biosynthetic gene cluster (*Le*Δ*hsaf*) significantly weakened the antagonistic phenotype against *C. krusei*, particularly in the non-contact system (Fig. 1F). Results show that for *L. enzymogenes*, wild-type *Le*YC36 exhibits a fold difference of approximately 0.49, indicating significant growth inhibition, while *Le*Δ*hsaf* has a fold difference of around 0.88 corresponding to only slight growth suppression. For *C. krusei*, the fold difference is 0.551 in contact co-culture with *Le*YC36 and 0.612 in non-contact co-culture with the same strain. With *Le*Δ*hsaf*, the fold difference of *C. krusei* is 0.870 in contact co-culture and approximately 0.918 in non-contact co-culture, confirming that *Le*YC36 possesses stronger antifungal activity against *C. krusei* than *Le*Δ*hsaf* (Fig. 1G). The attenuation of this antagonistic relationship indicates that, although other factors, such as the T6SS, may be involved in the direct contact co-culture process (5, 33), HSAF is the key effector driving bacteria-fungi antagonism, with its inhibitory effect being particularly pronounced under non-contact conditions.

Under non-contact conditions, the enhanced production of HSAF prompted us to hypothesize that diffusible fungal metabolites may serve as signaling cues mediating this cross-kingdom interaction. The extracted metabolites stimulated HSAF biosynthesis in a dose-dependent manner (Fig. S1). This result indicates that small-molecule metabolites secreted by *C. krusei* can act as potential chemical signaling cues that trigger HSAF production in *L. enzymogenes*, thereby mediating bacteria-fungi antagonism. Notably, HPLC analysis of the extracted *C. krusei* metabolites revealed that one of the key compounds was farnesol (Fig. 1H). Under our culture conditions, extracellular farnesol concentrations ranged from 33 to 48 μM (Fig. 1I; Fig. S2; Table S3). Farnesol, initially identified as a key quorum-sensing molecule in *Candida* species that inhibits hyphal development and biofilm formation (16, 17), was then investigated for its role in regulating HSAF production and mediating bacteria-fungi antagonism. Farnesol did not inhibit the growth of *Le*YC36 (Fig. S3A), but significantly enhanced the inhibitory activity of *Le*YC36 against *C. krusei* (Fig. 1I; Fig. S3B). In addition, different concentrations of farnesol also resulted in a dose-dependent increase in both HSAF production and the transcriptional levels of key biosynthetic genes in *Le*YC36 (Fig. 1J; Fig. S3C). These findings preliminarily support the hypothesis that farnesol secreted by *C. krusei* can also function as a cross-kingdom signaling molecule, enhancing HSAF biosynthesis in *Le*YC36 and thereby promoting antagonistic interactions during co-culture.

### A farnesol-mediated cross-kingdom in bacterial-fungal antagonism triggers HSAF biosynthesis

To further confirm farnesol’s role in cross-kingdom signaling, we blocked its biosynthesis in *C. krusei* to explore downstream effects on fungal physiology and interkingdom interactions. We used lovastatin, a specific HMG-CoA (3-hydroxy-3-methylglutaryl-coenzyme A) reductase inhibitor (34), which disrupts the mevalonate pathway to reduce production of farnesyl pyrophosphate, farnesol’s direct precursor, and ultimately suppresses farnesol secretion in *Candida*. When *Candida* cell density exceeds ~10⁶ CFU/mL, farnesol is actively secreted extracellularly via efflux pumps such as Cdr1. It inhibits hyphal formation, maintains yeast morphology, and suppresses biofilm matrix production by regulating morphogenesis (35, 36). We thus systematically quantified extracellular farnesol levels, fungal morphology, and biofilm formation in response to increasing lovastatin concentrations.

**Fig 2:**
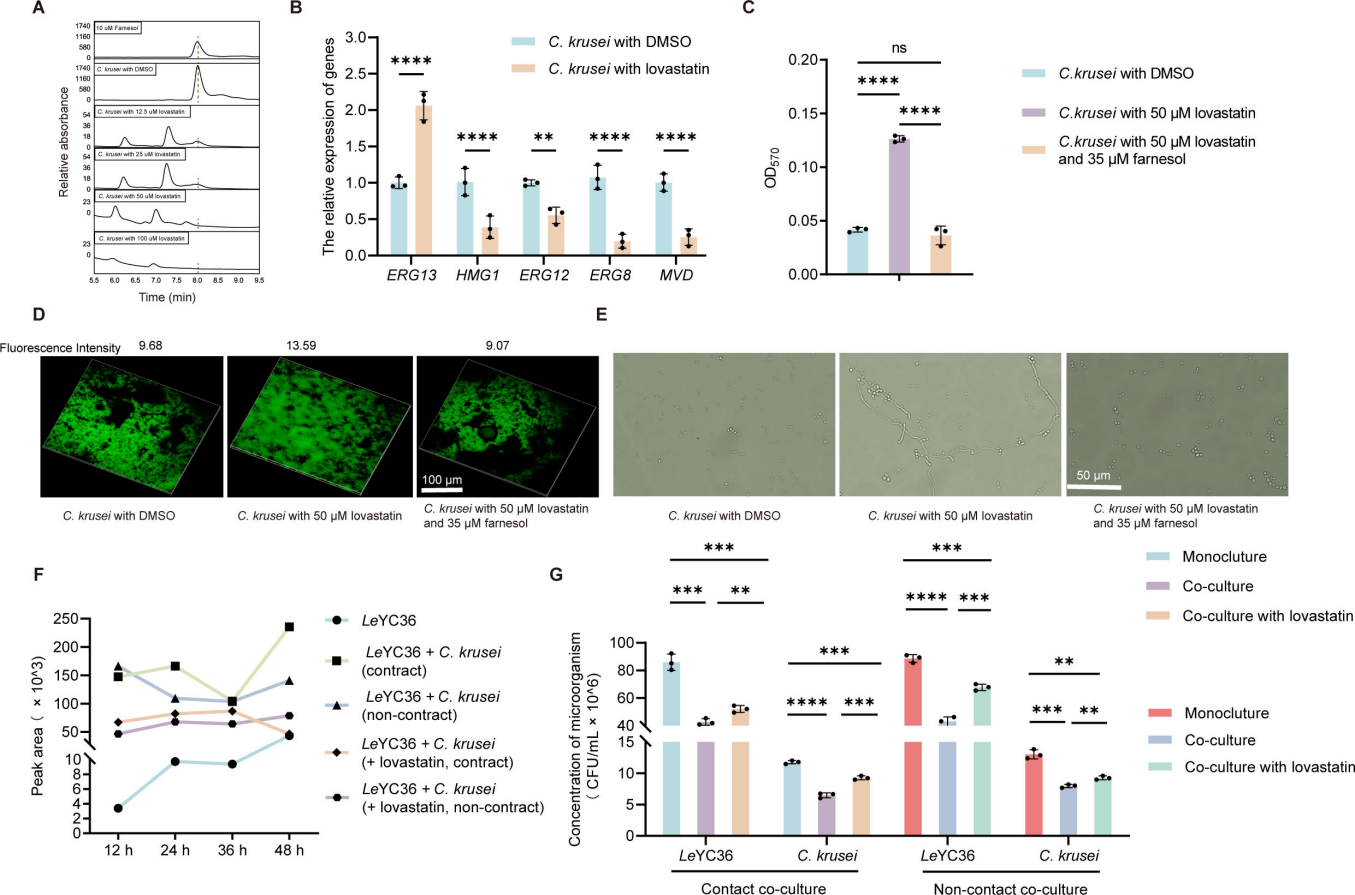
Effects of inhibiting farnesol biosynthesis on *C. krusei* farnesol metabolism and *Le*YC36 HSAF regulation during co-culture. (**A**) HPLC analysis of the effect of lovastatin addition on farnesol secretion by *C. krusei*. (**B**) Changes in the expression levels of key farnesol synthesis enzymes following lovastatin addition were determined by RT-qPCR. (**C**) Biofilm formation of *C. krusei* strains was quantified by crystal violet staining. (**D**) Visualization of biofilm alterations under various treatment conditions using confocal laser scanning microscopy. (**E**) Morphological characteristics of *C. krusei* under different treatments were observed by microscopy. (**F**) HPLC analysis of the HSAF production in *Le*YC36 following lovastatin treatment under the co-cultivation system. (**G**) Expression of the *hsaf* gene after lovastatin addition under the co-cultivation system. For panels B, C, and G, error bars indicate the SD of three replicates. Data presented as mean ± SD. For significance information, ns, not significant, **P value < 0.01, ***P value < 0.001, ****P value < 0.0001.

HPLC analysis revealed a dose-dependent reduction in extracellular farnesol, and supplementation with lovastatin at concentrations of 50 μM or higher completely abolished detectable farnesol production (Fig. 2A), confirming that lovastatin effectively inhibits farnesol biosynthesis in *C. krusei*. According to the farnesol biosynthesis pathway (37–39), *ERG13*, *HMG1*, *ERG12*, *ERG8*, and *MVD* play important roles in the farnesol synthesis process. Real-time PCR results showed that after treatment with lovastatin, *ERG13* in *C. krusei* was upregulated, whereas the other enzymes were downregulated (Fig. 2B). This indicates that lovastatin inhibits farnesol synthesis by suppressing HMG1, and the upregulation of *ERG13* may reflect a compensatory response to impaired farnesol synthesis.

Farnesol inhibits hyphal formation, maintains the yeast morphology, and suppresses biofilm matrix production by regulating morphogenesis (17, 40, 41). We therefore systematically quantified fungal morphology and biofilm formation in response to increasing lovastatin concentrations. Phenotypically, 50 μM lovastatin promoted *C. krusei* biofilm formation (Fig. S4A and B) and shifted its morphology from yeast-like to hyphal-like (Fig. S4C), effects reversed by supplementing 35 μM exogenous farnesol (Fig. 2C through E), confirming lovastatin-induced physiological changes in *C. krusei* were due to farnesol depletion. In addition, we further verified whether lovastatin directly affects the synthesis of antifungal substances in *Le*YC36. *Le*YC36 produces two major antifungal substances, HSAF and GluB (42). The experimental results demonstrated that lovastatin does not directly regulate the synthesis of HSAF and GluB, and we also found that it exerts no effect on the survival rates of *Le*YC36 and *C. krusei* (Fig. S5). This thus excludes the potential confounding effects of the drug itself on subsequent co-culture experiments.

To further clarify the role of farnesol in regulating HSAF production during bacterial-fungal interactions, 50 μM lovastatin was added to both contact and non-contact co-culture systems of *Le*YC36 and *C. krusei*. Although co-culture still resulted in upregulated HSAF gene expression and production compared to *Le*YC36 alone, the magnitude of this upregulation was markedly attenuated in the presence of lovastatin (Fig. 2F; Fig. S6). These findings suggest that farnesol plays a key, but not exclusive, role in mediating the fungal induction of HSAF biosynthesis. Additional signaling molecules secreted by *C. krusei*, such as tyrosol, which is another known quorum-sensing molecule in *Candida* (41), may also synergistically regulate HSAF synthesis. In addition, we further measured the biomass of co-cultures. In the presence of 50 μM lovastatin, the reduction in biomass of both *Le*YC36 and *C. krusei* was significantly alleviated compared to the untreated co-culture group, indicating a weakened antagonistic interaction (Fig. 2G). These results reflect the reduction in HSAF production, supporting its role as a central effector mediating bacterial-fungal antagonism, and suggesting that farnesol, together with other fungal metabolites, may modulate cross-kingdom interactions by regulating HSAF synthesis in *Le*YC36.

### Two-component system RcsB/C mediates farnesol sensing and HSAF biosynthesis in *L. enzymogenes*

**Fig 3:**
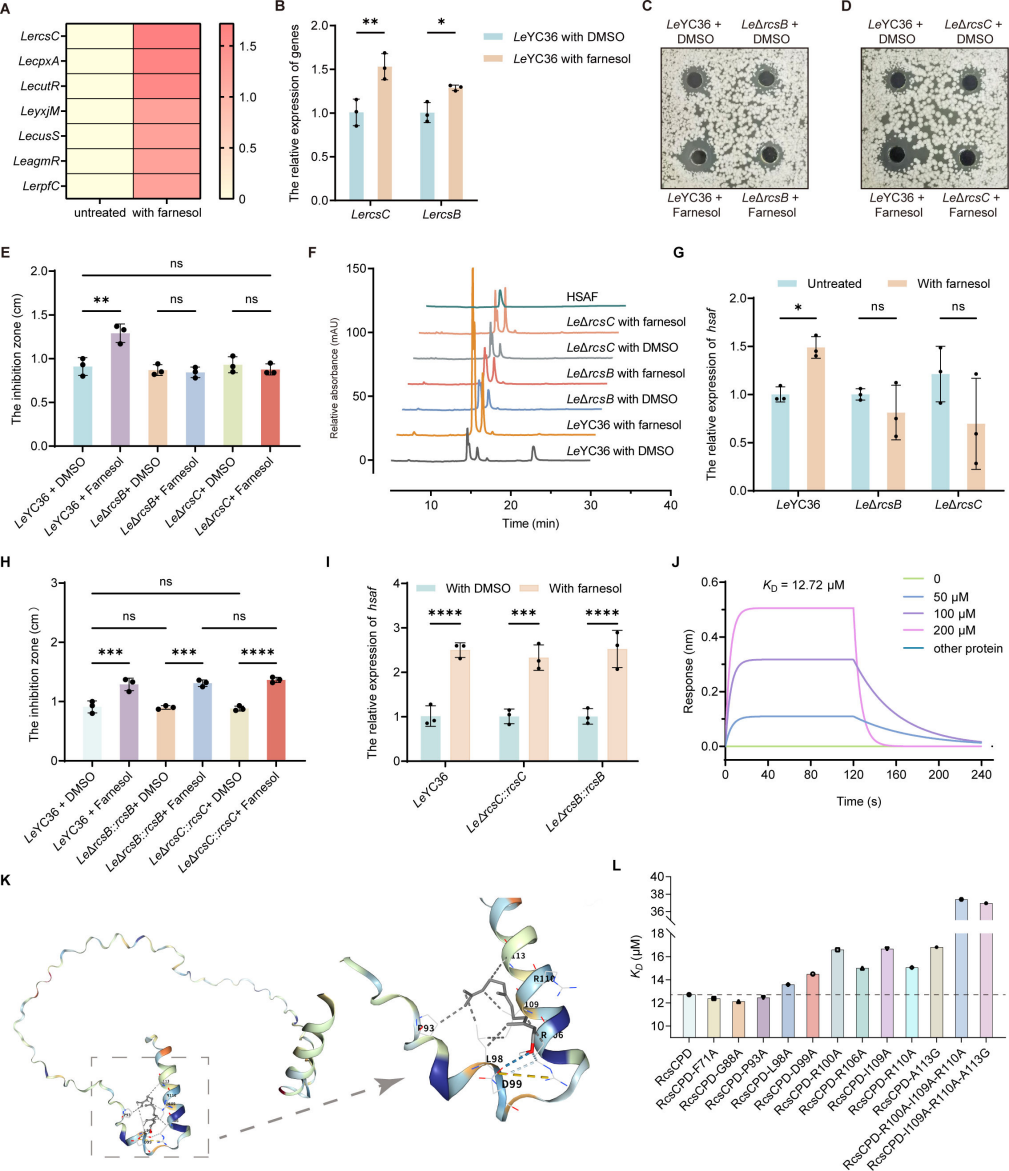
The two-component system RcsB/C senses farnesol and regulates HSAF biosynthesis and antifungal activity. (**A**) Upregulated two-component systems in *Le*YC36 under exogenous farnesol treatment. (**B**) Relative expression levels of *rcsB* and *rcsC* in *Le*YC36 upon exogenous farnesol addition were determined by RT-qPCR. (**C**) Antifungal effect of *Le*Δ*rcsB* against *C. krusei* following exogenous farnesol addition by the agar diffusion assay. (**D**) Antifungal effect of *Le*Δ*rcsC* against *C. krusei* following exogenous farnesol addition by the agar diffusion assay. (**E**) Statistical results of antifungal zones. (**F**) HPLC analysis of the HSAF production levels of *Le*Δ*rcsB* and *Le*Δ*rcsC* under farnesol treatment. (**G**) Relative expression level of HSAF biosynthetic genes in *Le*Δ*rcsB* and *Le*Δ*rcsC* with farnesol addition was determined by RT-qPCR. (**H**) Statistical data of antifungal zones of *rcsB*/*C* knockout-complemented strains. (**I**) Relative expression level of HSAF biosynthetic genes in rcs*B*/*C* knockout-complemented strains. (**J**) Biolayer interferometry (BLI) analysis of the binding between RcsC_PD_ and farnesol. (**K**) Key sites of RcsC_PD_ binding to farnesol predicted by AutoDock. (**L**) The *K_D_* values from the binding analysis of mutant RcsC_PD_ proteins with farnesol. Error bars indicate the SD of three replicates. Data presented as mean ± SD. For significance information, ns, not significant, *P value < 0.05, **P value < 0.01, ***P value < 0.001, ****P value < 0.0001.

Bacteria rely on highly conserved signal transduction systems to sense environmental cues and modulate physiological responses. TCSs, composed of a sensor histidine kinase and a cognate response regulator, are widely employed to convert external signals into intracellular regulatory outputs (22, 43–45) and regulate multiple phenotypes through phosphorylation cascades (46–48). To elucidate the signaling pathway responsible for farnesol-induced HSAF biosynthesis in *Le*YC36, we performed transcriptomic profiling under farnesol treatment. Among the differentially expressed TCS-related genes, the RcsB/C system was significantly upregulated and identified as a key candidate (Fig. 3A). This finding was further validated by real-time PCR (Fig. 3B; Fig. S7). Therefore, we further constructed *rcsB/C* deletion mutants and their complemented strains. In the *Le*Δ*rcsB* and *Le*Δ*rcsC* mutants, farnesol treatment failed to significantly enhance antifungal activity (Fig. 3C through E), HSAF production (Fig. 3F), or upregulation of HSAF-related gene expression (Fig. 3G). In addition, compared with the wild-type strain, exogenous farnesol did not enhance the survival rate of *C. krusei* in *Le*Δ*rcsB* and *Le*Δ*rcsC* (Fig. S8A). In contrast, the complemented strains restored farnesol-induced antifungal activity (Fig. 3H; Fig. S8B through D), HSAF gene expression (Fig. 3I), and biosynthesis (Fig. S8E). These results confirm that the TCS RcsB/C functions as a key regulatory factor involved in sensing farnesol and initiating HSAF biosynthesis.

To verify the farnesol regulatory pathway in *L. enzymogenes*, we performed domain analysis on the sensing protein RcsC (Fig. S9) and expressed the periplasmic domain of RcsC (RcsC_PD_). Biochemical characterization further demonstrated that RcsC_PD_ directly binds farnesol in a concentration-dependent manner, with a dissociation constant (*K*_D_) of 12.72 μM (Fig. 3J). This result confirms that RcsC can directly recognize farnesol via its periplasmic domain. The structure of the RcsC_PD_ protein was predicted using AlphaFold3 (49) (Fig. S10A), and molecular docking between RcsC_PD_ and farnesol was performed via AutoDock (50) to identify potential key farnesol-binding sites (Fig. 3K). Combined with conserved site analysis of RcsC_PD_ (Fig. S10B), ten amino acid residues were identified as candidates for mediating farnesol binding, including F71, G88, P93, L98, D99, R100, R106, I109, R110, and A113. Site-directed mutagenesis was performed on these residues, substituting A113 with glycine (A113G) and the remaining nine residues with alanine. Biolayer interferometry (BLI) assays of the purified mutant proteins revealed that the mutations L98A, D99A, R100A, R106A, I109A, R110A, and A113G significantly reduced the binding affinity of RcsC_PD_ to farnesol, with a concurrent increase in *K*_D_ values (Fig. 3L; Fig. S11). Based on the results of single-site mutant proteins, we further constructed multi-site mutants targeting residues that exhibited weakened binding ability to farnesol, generating two mutant proteins: R100A-I109A-R110A and I109A-R110A-A113G. Binding assays revealed that both multi-site mutants significantly reduced the binding capacity of RcsC_PD_ to farnesol (Fig. 3L). These findings support our hypothesis that these amino acids may achieve efficient binding through a cooperative network of hydrogen bonds-hydrophobic interactions-salt bridges. P93 and D99 are responsible for initial anchoring, R106 and R110 reinforce the interaction through both electrostatic and hydrophobic effects, I109 encapsulates the small molecule via the hydrophobic pocket, and A113 maintains the structural integrity of the pocket. This multi-mechanistic synergy ensures the stability of small-molecule binding. In summary, this result confirms that the periplasmic domain of RcsC can directly sense farnesol, with several key residues playing important roles in the sensing process.

### Farnesol-activated RcsC-RcsB-MarR2 signaling cascade drives HSAF biosynthesis in *L. enzymogenes*

**Fig 4:**
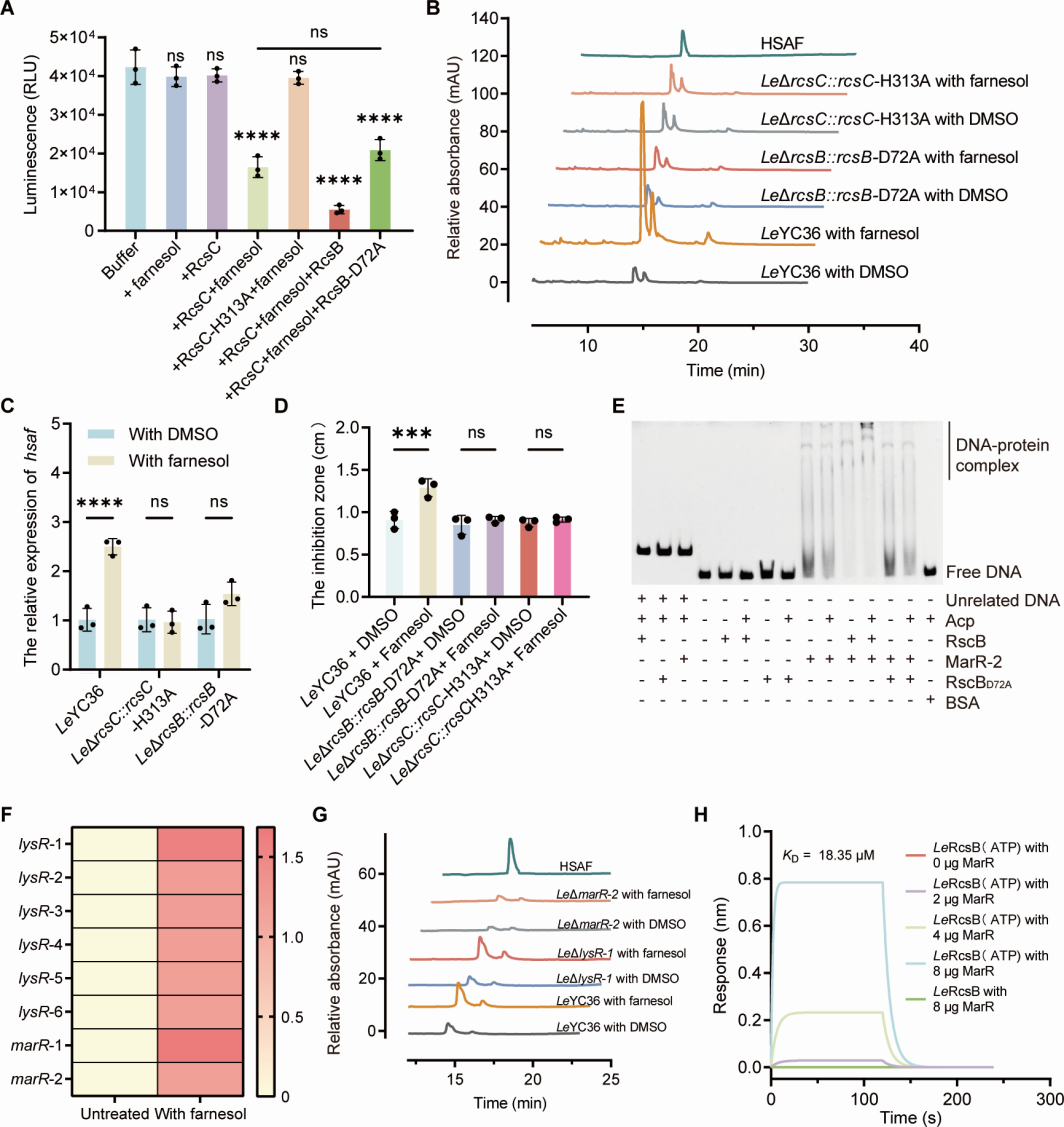
Farnesol-induced antifungal signaling mechanism and regulatory pathway driven by the RcsB/C TCS. (**A**) *In vitro* phosphotransfer assay revealing phosphate transfer from RcsC to RcsB. (**B**) HPLC analysis of the HSAF synthesis in *Le*Δ*rcsB::rcsB*-D72A and *Le*Δ*rcsC::rcsC*-H313A following farnesol addition. (**C**) Relative expression level of HSAF biosynthetic genes in *Le*Δ*rcsB::rcsB*-D72A and *Le*Δ*rcsC::rcsC*-H313A following farnesol addition was determined by RT-qPCR. (**D**) Antifungal ability of *Le*Δ*rcsB::rcsB*-D72A and *Le*Δ*rcsC::rcsC*-H313A following farnesol addition by the agar diffusion assay. (**E**) Electrophoretic mobility shift assays (EMSA) showing the binding of downstream regulators to the *hsaf* promoter. (**F**) Transcriptional levels of candidate downstream regulatory genes. (**G**) HSAF production in *Le*Δ*lysR-1* and *Le*Δ*marR-2* mutants upon farnesol induction. (**H**) The direct binding between RcsB and MarR-2 proteins is confirmed by BLI experiment. Error bars indicate the SD of three replicates. Data presented as mean ± SD. For significance information, ns, not significant, ***P value < 0.001, ****P value < 0.0001.

Based on the analysis of multiple sequence alignment, we identified the autophosphorylation site of RcsC (H313) (Fig. S12) and the phosphorylation site of RcsB (D72) (Fig. S13). Purified wild-type and mutant RcsB/C proteins were used in *in vitro* phosphotransfer assays (Fig. S14). We found that farnesol enhanced RcsC autophosphorylation and further promoted transfer of the phosphate group to the response regulator RcsB, while this process is abolished in the H313A and D72A mutants (Fig. 4A), confirming that H313 and D72 are critical residues required for TCS activity. Furthermore, we constructed *in situ* point mutant strains, *Le*Δ*rcsB::rcsB*-D72A and *Le*Δ*rcsC::rcsC*-H313A. Real-time PCR and HPLC analyses showed that exogenous farnesol treatment no longer induced significant upregulation of HSAF-related gene expression or HSAF production in these mutants (Fig. 4B and C). In addition, farnesol treatment no longer enhanced the antifungal activity of the *in situ* mutant strains (Fig. 4D; Fig. S15). Together, these experiments validate the RcsB/C-driven signaling transduction mechanism, in which sensing of farnesol triggers RcsC autophosphorylation at H313 and subsequent phosphate transfer to RcsB at D72, a process essential for downstream gene regulation.

**Fig 5:**
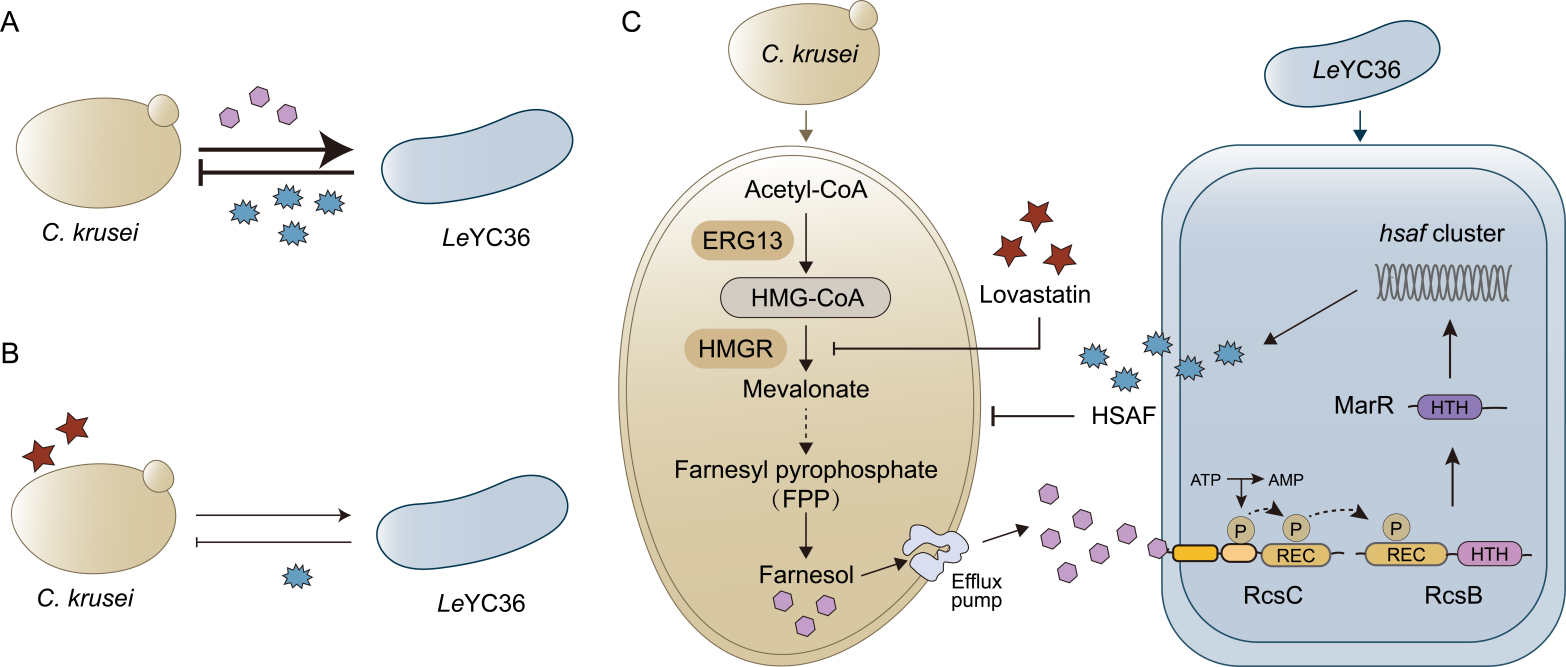
Molecular mechanism and regulatory pathway underlying farnesol-mediated antagonistic interaction between *L. enzymogenes* and *C. krusei*. The antagonistic relationship between *Le*YC36 and *C. krusei* under normal conditions (**A**)and under conditions of farnesol secretion inhibition (**B**).The thickness of the lines represents the strength of the antagonistic relationship. (**C**) Regulatory pathway of farnesol-induced antifungal activity driven by RcsB/C in *Le*YC36. *C. krusei* produces the quorum-sensing molecule farnesol for its own communication, which can be sensed by the RcsB/C in *L. enzymogenes*. Upon farnesol recognition, RcsC transfers the phosphate groups to RcsB, which subsequently enhances the binding affinity of the downstream transcription factor MarR-2 to the *hsaf* promoter, thereby activating transcription of the HSAF biosynthetic gene cluster. The resulting increase in HSAF production suppresses fungal growth, constituting a feedback antagonistic response.

To determine whether RcsB can directly regulate downstream HSAF gene expression, we performed electrophoretic mobility shift assays (EMSA) using the predicted promoter sequences of the HSAF gene cluster. Neither unphosphorylated nor phosphorylated RcsB formed DNA-protein complexes, indicating that RcsB does not directly regulate HSAF synthesis but likely acts through downstream transcription factors (Fig. 4E). Based on previous reports linking LysR- and MarR-family transcription factors to HSAF regulation (51), we screened differentially expressed genes under farnesol treatment, focusing on *lysR* and *marR* homologs in *Le*YC36 (Fig. 4F), and identified *lysR-1* and *marR-2* as candidate genes (Fig. S16). Deletion of *lysR*-1 did not affect farnesol-induced HSAF upregulation, whereas deletion of *marR*-2 abolished both basal and farnesol-induced HSAF production (Fig. 4G). Consistent with this, *marR*-2 expression was significantly downregulated in *Le*Δ*rcsB* and *Le*Δ*rcsC* compared to wild type under farnesol treatment, indicating that MarR-2 functions as a critical downstream regulator in the RcsB/C pathway (Fig. S16B). EMSA results further demonstrated that MarR-2 directly binds the promoter of the HSAF biosynthetic gene cluster, and the presence of phosphorylated RcsB significantly enhanced MarR-2 binding, whereas the RcsB_D72A_ mutant had no effect (Fig. 4E). This finding reveals that phosphorylation of RcsB potentiates the regulatory activity of MarR-2 and drives HSAF biosynthesis (Fig. 4E). To verify whether the phosphorylated RcsB can interact with MarR-2, we performed protein–protein interaction validation using BLI. The results showed that only phosphorylated RcsB could bind to MarR-2, with a *K*_D_ of 18.35 μM (Fig. 4H). Collectively, these results demonstrate that farnesol can act as a signaling molecule and play an important role in bacterial–fungal interactions, and blocking farnesol biosynthesis also attenuates the antagonistic relationship between these bacteria and fungi (Fig. 5A and B). We also elucidated the molecular mechanism and regulatory pathway by which farnesol modulates HSAF biosynthesis in *L. enzymogenes*. This regulatory cascade, farnesol-RcsC-RcsB-MarR-2-HSAF, demonstrates how *L. enzymogenes* “hijacks” and senses fungal-derived chemical signals from a competing organism to amplify antifungal metabolite production(Fig. 5C). These findings highlight the complexity of cross-kingdom chemical communication and provide new mechanistic insights into bacterial-fungal antagonism.

## DISCUSSION

The stability of microbial communities is influenced by diverse chemical signals, which mediate communication that promotes cooperation, competition, antagonism, and niche partitioning among community members (13). Previous studies have reported various strategies by which fungi and bacteria interact through signaling molecules (52). For example, *Candida albicans* produces farnesol, which can reduce bacterial toxicity toward the fungus by decreasing *Pseudomonas* quinolone signal-mediated virulence (53, 54). In contrast, in the oral cavity, *Streptococcus gordonii* engages in a mutualistic interaction with *C. albicans*, where signaling molecules facilitate coexistence and promote fungal biofilm development (55, 56). Our study uncovers a novel ecological strategy employed during bacterial-fungal interactions: rather than being inhibited by the QS molecule farnesol derived from the *C. krusei*, *L. enzymogenes* “hijacks” this signal, turning the opponent’s own cues against them to reinforce its antifungal capacity. Specifically, during co-culture, farnesol secreted by *C. krusei* is sensed by the TCS RcsB/C in *L. enzymogenes*. Although activated RcsB does not directly control antifungal metabolite synthesis, it enhances the activity of the downstream transcriptional regulator MarR-2, thereby indirectly promoting the expression of genes responsible for HSAF biosynthesis. This newly identified regulatory cascade illustrates how *L. enzymogenes* integrates external fungal signals into its regulatory network to amplify antifungal metabolite production. Notably, while the TCS RcsB/C is characterized in enteric bacteria for cell envelope stress responses and biofilm regulation (57, 58), its role in fungal signal sensing has not been reported, revealing an important missing link in RcsB/C-mediated cross-kingdom communication. By elucidating this complex mechanism, our work identifies key amino acid residues involved in farnesol signal recognition, establishes a direct link between fungal QS molecule farnesol and bacterial TCS RcsB/C, and provides new insights into interkingdom chemical communication. Moreover, although farnesol has been extensively studied in *Candida* species as a QS molecule, our findings highlight its additional role as a cross-kingdom signal that can be recognized by bacteria to activate secondary metabolism and modulate cross-kingdom antagonism. This expands the functional scope of farnesol from intra-kingdom regulation to cross-kingdom signaling.

Although farnesol plays a critical role in this pathway, our results also demonstrate that it is not the sole determinant of HSAF induction. This conclusion is supported by the inhibition of fungal farnesol biosynthesis by lovastatin. This study demonstrates that lovastatin itself does not directly affect the biosynthesis of the major antifungal compounds HSAF and GluB in *Le*YC36. The observed changes during strain interaction are mainly attributed to the regulatory effects of small-molecule metabolites secreted by *C. krusei*, such as farnesol. This finding provides key evidence for identifying the targets of lovastatin in cross-kingdom interactions. It further confirms that farnesol is an important signaling molecule mediating the regulation between *C. krusei* and *Le*YC36. This study has focused on the verification of core antifungal compounds. The potential effects of lovastatin on the physiological status, secretion system, or uncharacterized antifungal pathways of *Le*YC36 cannot be fully excluded. These potential regulatory mechanisms and signaling pathways remain unclear. They need to be systematically explored in future studies through multi-omics analysis and molecular interaction verification. Further exploration of these unknown mechanisms will promote a more comprehensive understanding of the regulatory role of lovastatin in microbial interactions. It will also provide new insights and theoretical support for dissecting the complex network of cross-kingdom interactions between *Lysobacter enzymogenes* and pathogenic fungi. Additionally, in the presence of lovastatin, extracellular farnesol levels decreased, leading to a reduced but not abolished upregulation of HSAF. This result indicates that additional fungal metabolites may also be involved in the antagonistic interaction between *L. enzymogenes* and *C. krusei*. Tyrosol, another well-characterized QS molecule in *Candida* species (19, 59, 60), is a reasonable candidate. Beyond tyrosol, a range of fungal secondary metabolites and volatile organic compounds may act in synergy with farnesol to regulate bacterial metabolite production (18). Thus, HSAF regulation in *L. enzymogenes* is likely governed by multiple fungal-derived signals rather than a single molecule, pointing to a complex regulatory system that enables the bacterium to gain a competitive advantage during antagonism with fungi. Integrating metabolomic analysis of fungal secretions, assessing combinatorial signaling cues, and analyzing receptor mutant analysis in *L. enzymogenes* is essential for identifying these additional signals and advancing our understanding of cross-kingdom microbial interactions. Beyond *Candida*, we also extracted metabolites from multiple fungi, including *Aspergillus niger*, *Aspergillus fumigatus*, *Penicillium* spp., *Rhizopus* spp., and *Fusarium oxysporum*. These metabolites also increased HSAF production to varying extents (Fig. S17). These findings suggest that *L. enzymogenes* can detect a broad spectrum of extracellular metabolites across diverse fungal taxa, exploiting chemical information from competitors to optimize resource allocation and enhance survival. In natural habitats such as soil and plant surfaces, where multiple fungal species coexist and secrete diverse metabolites (3, 4, 18), this ability allows *L. enzymogenes* to selectively induce HSAF synthesis when encountering fungal competitors. This targeted induction may contribute to maintaining microbial population balance and suppressing fungal overgrowth (60, 61). This also provides promising opportunities for the development of novel biocontrol strategies, in which bacterial sensing of fungal signals can be harnessed to inhibit plant fungal pathogens in a more targeted manner.

Future research should aim to systematically identify additional fungal-derived metabolites that contribute to regulating HSAF and to characterize their specific bacterial receptors and downstream regulators. Compared with pharmacological inhibition, deletion of key genes in the fungal farnesol biosynthetic pathway would provide more direct evidence of its functional role. Moreover, further structural and biochemical analyses to determine the molecular basis of farnesol binding to RcsC will help elucidate the mechanisms of ligand recognition and signal perception. Finally, community-level analyses under soil- or plant-associated conditions will be essential to elucidate how these regulatory pathways influence microbial community assembly and stability. In summary, our findings reveal a novel bacterial-fungal antagonistic strategy in which *L. enzymogenes* hijacks a fungal QS molecule to trigger antifungal metabolite production via a TCS and downstream transcriptional regulators. This strategy highlights the functional versatility of quorum-sensing molecules. By extending the role of farnesol into cross-kingdom communication, this work emphasizes chemical signaling as a dynamic driver of interkingdom interactions, provides new perspectives on bacterial-fungal antagonism, and suggests promising directions for harnessing microbial communication in biocontrol applications.

## MATERIALS AND METHODS

### Bacterial strains, plasmids, general methods, and materials

*Lysobacter enzymogenes* YC36 (*Le*YC36) and its mutant strains were cultured at 28°C in 40% tryptic soy broth (TSB). *Candida krusei* and *Escherichia coli* strains were grown on SDA at 28°C and in LB at 37°C, respectively. *E. coli* S17-1 λpir and BL21(DE3) were used for conjugation and protein expression. Bacterial-fungal co-culture experiments used 10% TSB to simulate nutrient-limited conditions. A full list of strains and plasmids is provided in Table S1.

Reagents for molecular biology and biochemistry were sourced from Sparkjade (China), Takara (Japan), Yeasen (China), and Tsingke (China). Farnesol was purchased from Aladdin (China). Primer synthesis and DNA sequencing were performed by Tsingke (China), with primer sequences listed in Table S2. All molecular techniques followed established protocols from previous publications (62).

### Bacterial-fungal co-culture

Co-culture of *Le*YC36 and *C. krusei* was initiated by spotting 10 μL of each microorganism (OD₆₀₀ adjusted to 1.0) onto 3 mL of solid 10% TSB medium in a 6-well plate. For direct interaction, the strains were inoculated 0.8 cm apart. To separate physical contact, a 0.22 μm sterile filter membrane was placed between the inoculation sites. Samples were collected every 12 h for RNA extraction and HSAF quantification. After 48 h, biomass was determined by dilution plating on selective media (LB with kanamycin for *Le*YC36, SDA for *C. krusei*).

### Extraction and detection of fungal metabolite farnesol

*C. krusei* was cultured in PDB at 28°C for 48 h. The supernatant was obtained by centrifugation and then extracted with an equal volume of ethyl acetate. The organic phase was dried, redissolved in methanol, and analyzed by HPLC (Hitachi Primaide; YMC-Pack Pro C18 column) at 210 nm. This HPLC detection method was slightly modified based on previously reported studies (63). Isocratic elution was performed with 80% acetonitrile (containing 0.1% formic acid) and 20% water (containing 0.1% formic acid) at a flow rate of 1 mL/min.

### Extraction and detection of HSAF

HSAF extraction and detection were performed as previously described with minor modifications (64, 65). After 48 h of culture, the bacterial suspension was centrifuged. A 3 mL aliquot of the supernatant was supplemented with 0.5 g of CaCl₂ and extracted with an equal volume of ethyl acetate. The dried extract was redissolved in methanol and analyzed by HPLC. Detection was performed at 320 nm using a gradient elution of mobile phase B (acetonitrile with 0.1% formic acid) in A (water with 0.1% formic acid) at a flow rate of 1 mL/min. The gradient program was as follows: 20%–35% B (0–5 min), 35%–75% B (5–17 min), 75%–90% B (17–27 min), and 100% B (27–30 min).

### Detection of biofilm synthesis

A 96-well plate was used, with each well containing 200 μL of 100% TSB medium supplemented with 1% *C. krusei* (OD₆₀₀ = 0.137). Lovastatin (Macklin, China) was added to final concentrations of 12.5, 25, 50, and 100 μM, and the plate was incubated at 28°C for 12 h. The method was performed as described previously (42). After crystal violet staining, the OD₅₇₀ was measured using a microplate reader (BioTek, USA).

### *C. krusei* biofilm assay by FITC staining

The assay method for biofilm formation was performed as described by Jian et al. (66). *C. krusei* with an OD₆₀₀ of 0.137 was inoculated into full-strength TSB medium at a 1% inoculum ratio. Sterilized coverslips were placed in 6-well plates, and 2 mL of the fungal suspension was added to each well, followed by static incubation at 28°C for 12 h. Subsequently, the fungal suspension was gently washed off with PBS, and the biofilms on the coverslips were stained with 1 mg/mL FITC for 10 min. After staining, the coverslips were gently rinsed three times with PBS and then observed immediately under a confocal laser scanning microscope.

### Agar diffusion assay

Strain preparation was performed according to the strain activation procedure described in the detection of the inhibitory activity of fermentation broth supernatant against *C. krusei*. The OD₆₀₀ value of *C. krusei* was adjusted to 0.1. A volume of 100 μL of the prepared *C. krusei* suspension was spread onto SDA medium. Wells were punched into the medium. A 20 μL of *Le*YC36 supernatant from different experimental groups was added to each well. The plates were air-dried and then incubated in a 28°C incubator for 1 day. The sizes of inhibition zones were subsequently observed and measured.

### Detection of the inhibitory activity of fermentation broth supernatant against *C. krusei*

*Le*YC36 was inoculated into 40% TSB at a 1% inoculation volume. Farnesol was added to the experimental group to a final concentration of 50 μM. An equal volume of DMSO was added to the control group. Cultures were incubated at 28°C for 48 h. Supernatant was collected by centrifugation. *C. krusei* was cultured in PDB medium at 28°C for 24 h. OD_600_ value was adjusted to 0.1 using an ultraviolet spectrophotometer. *C. krusei* suspension with OD_600_ = 0.1 was mixed with *Le*YC36 supernatant at a 1:2 ratio. An equal volume of 40% TSB was added to the control group. Mixtures were co-cultured at 28°C for 24 h. Serial dilutions were performed. Dilutions were spread on SDA medium. Plates were incubated at 28°C for 2–3 days. The number of single colonies was counted.

### Construction of in-frame deletion and *in situ* site-directed mutant strains

Gene deletion mutants in *Le*YC36 (GCF_005954665.1) were constructed using overlapping PCR to fuse the upstream and downstream homologous arms of the target gene. The resulting fragment was cloned into a suicide vector, and the recombinant plasmid was extracted and purified (SPARKeasy Mini Plasmid Ultra-Fast Kit, Shandong Sparkjade Biotechnology Co., Ltd.). Homologous recombination was then performed as described previously (62, 67), with mutants obtained through double selection on antibiotic and 15% sucrose plates.

*In situ* site-directed mutants of the RcsB/C system were generated by first identifying conserved phosphorylation sites (RcsC autophosphorylation and RcsB phosphorylation sites) via sequence alignment (Fig. S12 and S13). Using the corresponding deletion mutants as parental strains, specific primers were designed. The mutants were then obtained through recombinant plasmid construction and homologous recombination.

### Transcriptome analysis

Transcriptome analysis of *Le*YC36 treated with DMSO or farnesol was performed by Scientsgene (Qingdao, China). RNA quality and concentration were determined using a Bioanalyzer 2100 (Agilent, USA) and NanoDrop 2000 (Thermo Scientific, USA), respectively. RNA-seq libraries were constructed using an Illumina RNA Preparation Kit and sequenced on the Illumina HiSeq platform. Raw data were processed using SeqPrep, Sickle, and Rockhopper.

### Detection of gene transcription levels

Total RNA was extracted from *Le*YC36 and *C. krusei* under monoculture, contact, and non-contact co-culture conditions, as well as from various treatment groups, using commercial kits (MolPure for bacteria and TRIeasy for fungi, YEASEN, China). Subsequently, 200 ng of RNA was reverse-transcribed into cDNA (All-In-One 5× RT MasterMix, abm, Canada). Real-time PCR was performed in a 20 μL reaction system containing Hieff qPCR SYBR Green Master Mix (High Rox Plus, YEASEN, China), using the 16S rRNA gene as an internal reference. Gene expression levels were calculated via the 2^(−ΔΔCt)^ method. All primers are listed in Table S2.

### Construction of mutant proteins

The amino acid sequence of RcsC_PD_ from *Le*YC36 was retrieved, and its three-dimensional structure was predicted using AlphaFold3 with default parameters. The top-ranked model was selected, and following the removal of water molecules and loop refinement, it was used for molecular docking. The farnesol structure was obtained from PubChem. Molecular docking was performed using AutoDock after preparing both the protein (adding hydrogens, assigning Gasteiger charges) and the ligand. The top 10 conformations, ranked by binding free energy, were analyzed to identify key farnesol-interacting residues, which guided the design of site-directed mutagenesis primers.

### Protein expression and purification

The construction and purification methods for protein expression strains were performed according to previous methods (62). The RcsB, RcsB_D72A_, RcsC_PD_, and MarR-2 proteins were cloned into the pET-28a(+) plasmid, while the RcsC and RcsC_H313A_ proteins were cloned into the pET-19b plasmid. Cultivate the expression strain at 37°C and 200 rpm. After reaching the logarithmic phase (OD₆₀₀ = 0.6–0.8), add isopropyl β-D-thiogalactopyranoside to a final concentration of 0.5 mM, and incubate at 16°C and 200 rpm for 16 h. Centrifuge at 6,500 rpm and 4°C for 15 min, then discard the supernatant. For protein expression strains containing the pET-28a(+) plasmid, resuspend the cells in 1/10 cell volume of Binding Buffer (20 mM Tris-HCl, 0.5 M NaCl, pH 8.0) and use an ultrasonic cell disruptor (JY92-IIN, Scientz, China) to lyse the cells (300 W, ultrasonication for 5 s, pause for 5 s, repeated for 30 min). Centrifuge at 12,000 rpm at 4°C for 15 min, collect the supernatant, and filter through a 0.22 μm membrane. For membrane proteins, resuspend the cells in 1/10 cell volume of lysis buffer (40 mM Tris-HCl, pH 8.0, 200 mM NaCl, 2 mM EDTA, 2 mM DTT, 0.1 mM PMSF, 10% glycerol) and disrupt using a high-pressure cell disruptor (Union-Biotech, China). Centrifuge at 12,000 rpm at 4°C for 1 h, remove cell debris, and collect the supernatant. Centrifuge at 39,000 rpm at 4°C for 1 h using an ultracentrifuge, discard the supernatant, collect the membrane fragments, and resuspend in lysis buffer (40 mM Tris-HCl, pH 8.0, 200 mM NaCl, 0.5 mM DTT, 10% DDM, 10% glycerol) to resuspend the membrane, and incubate at 4°C overnight to solubilize the membrane. Centrifuge at 39,000 rpm and 4°C for 30 min and collect the supernatant.

Perform protein purification using Ni-NTA affinity chromatography. Elute sequentially with imidazole solutions of varying concentrations (prepared using Binding Buffer, pH 8.0, 10/20/50/75/100/250/500 mM imidazole) to elute, and membrane proteins were eluted with lysis buffer (40 mM Tris-HCl, pH 8.0, 200 mM NaCl, 0.5 mM DTT, 10% DDM, 10% glycerol, 10/50/100/250/500 mM imidazole) elution, followed by detection using sodium dodecyl sulfate polyacrylamide gel electrophoresis.

### Biolayer interferometry

The method was modified based on previous methods (68, 69). Protein-small molecule and protein–protein interactions were analyzed on an Octet R8 system (Sartorius, Germany) at 25°C using PBST as the running buffer. For farnesol binding, purified His-RcsC_PD_ (wild-type and key site mutants) was immobilized on Ni-NTA biosensors (Sartorius, Germany) and associated with farnesol (0–200 μM). For protein interaction, phosphorylated or unphosphorylated His-RcsB was immobilized, and gradient concentrations (2, 4, 8 μg) of TEV-cleaved MarR-2 were used as analytes. All binding kinetics were recorded, and the dissociation constant (*Kᴅ*) was calculated using Octet Analysis Studio 13.0.

### *In vitro* autophosphorylation and phosphotransfer assays

Autophosphorylation and phosphorylation of TCS are carried out by consuming ATP (70). By measuring the remaining ATP content in the reaction system using luciferase, it is possible to indirectly determine whether protein autophosphorylation and phosphate group transfer have occurred (71). Kinase activity was assessed by quantifying the residual ATP in the reaction system using the Kinase-Lumi Plus Luminescent Kinase Assay Kit (Beyotime, China). The assay principle is based on the luciferase-catalyzed oxidation of luciferin, which consumes ATP and produces a proportional luminescent signal. Reactions were conducted in a black 96-well plate with a total volume of 50 μL, containing 10 μL of 5× phosphate kinase buffer and 2 μL of 0.5 mM ATP. Test groups were supplemented with farnesol, purified BL21-His-RcsC protein, a combination thereof, or various mutant proteins to investigate the phosphate transfer pathway.

### Electrophoretic mobility shift assay

This method was modified from a reported protocol (72). The HSAF promoter fragment was predicted using BPROM and BDGP, amplified by PCR, and gel-purified. EMSA was performed in a 10 μL reaction system containing purified protein (His-RcsB, His-RcsB-D72A, or His-MarR-2), DNA fragment, and 5× binding buffer. For phosphorylation assays, 600 mM acetyl phosphate was added. After 15 min incubation at 25°C, samples were resolved on a 6% native polyacrylamide gel (0.5× TBE, 110 V). Gels were stained with YeaRed nucleic acid dye (Yeasen, China) and visualized using a gel imaging system (JY04S-3E, Junyi, China).

### Gene identification and conserved site analysis

RcsC and RcsB were identified via BLASTP (NCBI database). BLASTP against SwissProt showed high homology with *E. coli* K-12 RcsB/C: RcsB (39.52% identity, 92% coverage, e-value = 8E-46; P0DMC7.1); RcsC (33.33% identity, 56% coverage, e-value = 8E-68; P0DMC5.1), thus named *Le*RcsB/*Le*RcsC.

Protein domains were predicted using SMART (73) and DeepTMHMM (74). SMART analysis revealed a MarR family domain in MarR-2, hence the name.

RcsB and RcsC are core functional components of the two-component system in *L. enzymogenes* YC36. The RcsC protein corresponds to the internal locus tag FE772_13200 of the strain, consisting of 876 amino acid residues with a theoretical molecular weight of 94.25 kDa. The RcsB protein corresponds to the internal locus tag FE772_13205 of the strain, consisting of 228 amino acid residues with a theoretical molecular weight of 24.08 kDa.

### Statistical analysis

Statistical analyses were performed using Origin 2019 and GraphPad Prism 9.5. Differences between biological replicates were analyzed by two-tailed Student’s *t*-test or ANOVA with Tukey’s multiple comparisons test. Student’s *t*-test is used to compare differences in means between only two groups of independent or paired samples, while ANOVA followed by Tukey’s multiple comparisons test is applied to three or more groups of independent samples, which first tests the overall differences among groups and then further identifies the specific differences between any two groups. All data were derived from at least three independent replicates. Statistical significance was denoted as follows: ns (not significant), *P < 0.05, **P < 0.01, *** *P* < 0.001, **** *P* < 0.0001.

## Data Availability

All data used in this study are available in the article and the supplemental material.

## SUPPLEMENTAL MATERIAL

Supplemental material aem.00304-26-s0001.pdf Fig. S1 to S17; Tables S1 to S3.

ASM does not own the copyrights to Supplemental Material that may be linked to, or accessed through, an article. The authors have granted ASM a non-exclusive, world-wide license to publish the Supplemental Material files. Please contact the corresponding author directly for reuse.

## References

[1] Stubbusch AKM, Peaudecerf FJ, Lee KS, Paoli L, Schwartzman J, Stocker R, Basler M, Schubert OT, Ackermann M, Magnabosco C, D’Souza GG. 2025. Antagonism as a foraging strategy in microbial communities. Science 388:1214–1217. doi:10.1126/science.adr8286 40504916

[2] García-Bayona L, Comstock LE. 2018. Bacterial antagonism in host-associated microbial communities. Science 361:eaat2456. doi:10.1126/science.aat2456 30237322

[3] Deveau A, Bonito G, Uehling J, Paoletti M, Becker M, Bindschedler S, Hacquard S, Hervé V, Labbé J, Lastovetsky OA, Mieszkin S, Millet LJ, Vajna B, Junier P, Bonfante P, Krom BP, Olsson S, van Elsas JD, Wick LY. 2018. Bacterial-fungal interactions: ecology, mechanisms and challenges. FEMS Microbiol Rev 42:335–352. doi:10.1093/femsre/fuy008 29471481

[4] Wagg C, Schlaeppi K, Banerjee S, Kuramae EE, van der Heijden MGA. 2019. Fungal-bacterial diversity and microbiome complexity predict ecosystem functioning. Nat Commun 10:4841. doi:10.1038/s41467-019-12798-y 31649246 PMC6813331

[5] Lin L, Shen D, Shao X, Yang Y, Li L, Zhong C, Jiang J, Wang M, Qian G. 2025. Soil microbiome bacteria protect plants against filamentous fungal infections via intercellular contacts. Proc Natl Acad Sci USA 122:e2418766122. doi:10.1073/pnas.2418766122 39813250 PMC11762177

[6] de Menezes AB, Richardson AE, Thrall PH. 2017. Linking fungal-bacterial co-occurrences to soil ecosystem function. Curr Opin Microbiol 37:135–141. doi:10.1016/j.mib.2017.06.006 28692866

[7] Grossart H-P, Van den Wyngaert S, Kagami M, Wurzbacher C, Cunliffe M, Rojas-Jimenez K. 2019. Fungi in aquatic ecosystems. Nat Rev Microbiol 17:339–354. doi:10.1038/s41579-019-0175-8 30872817

[8] Peterson SB, Bertolli SK, Mougous JD. 2020. The central role of interbacterial antagonism in bacterial life. Curr Biol 30:R1203–R1214. doi:10.1016/j.cub.2020.06.103 33022265 PMC7595158

[9] Klein TA, Ahmad S, Whitney JC. 2020. Contact-dependent interbacterial antagonism mediated by protein secretion machines. Trends Microbiol 28:387–400. doi:10.1016/j.tim.2020.01.003 32298616

[10] Granato ET, Meiller-Legrand TA, Foster KR. 2019. The evolution and ecology of bacterial warfare. Curr Biol 29:R521–R537. doi:10.1016/j.cub.2019.04.024 31163166

[11] Liu Y, Zuo Y, Li C, Fu P, He X, Wang Z, Li Y, Wan C, Wang Y, Wang Y, Zhu L, Shen X. 2025. Activation of an antifungal pathway in Yersinia pseudotuberculosis by chitin-receptor-mediated fungal recognition. Curr Biol 35:2672–2683. doi:10.1016/j.cub.2025.04.072 40403720

[12] Mirtaleb MS, Bakhshandeh B, Mohammadipanah F, Seyed Shirazi SR, Mobashery AR. 2025. Bacterial and fungal quorum sensing interactions with human cells; mechanisms and potential therapeutical applications. Microb Pathog 207:107925. doi:10.1016/j.micpath.2025.107925 40714271

[13] Khalid S, Keller NP. 2021. Chemical signals driving bacterial-fungal interactions. Environ Microbiol 23:1334–1347. doi:10.1111/1462-2920.15410 33511714

[14] Zheng H, Kim J, Liew M, Yan JK, Herrera O, Bok JW, Kelleher NL, Keller NP, Wang Y. 2015. Redox metabolites signal polymicrobial biofilm development via the NapA oxidative stress cascade in Aspergillus. Curr Biol 25:29–37. doi:10.1016/j.cub.2014.11.018 25532893 PMC4286458

[15] Schmidt R, Etalo DW, de Jager V, Gerards S, Zweers H, de Boer W, Garbeva P. 2015. Microbial small talk: volatiles in fungal-bacterial interactions. Front Microbiol 6:1495. doi:10.3389/fmicb.2015.01495 26779150 PMC4700264

[16] Nickerson KW, Gutzmann DJ, Boone CHT, Pathirana RU, Atkin AL. 2024. Physiological adventures in Candida albicans: farnesol and ubiquinones. Microbiol Mol Biol Rev 88:e0008122. doi:10.1128/mmbr.00081-22 38436263 PMC10966945

[17] Polke M, Leonhardt I, Kurzai O, Jacobsen ID. 2018. Farnesol signalling in Candida albicans - more than just communication. Crit Rev Microbiol 44:230–243. doi:10.1080/1040841X.2017.1337711 28609183

[18] Padder SA, Prasad R, Shah AH. 2018. Quorum sensing: a less known mode of communication among fungi. Microbiol Res 210:51–58. doi:10.1016/j.micres.2018.03.007 29625658

[19] Rodrigues CF, Černáková L. 2020. Farnesol and tyrosol: secondary metabolites with a crucial quorum-sensing role in Candida biofilm development. Genes (Basel) 11:444. doi:10.3390/genes11040444 32325685 PMC7231263

[20] Moreno-Gámez S, Hochberg ME, van Doorn GS. 2023. Quorum sensing as a mechanism to harness the wisdom of the crowds. Nat Commun 14:3415. doi:10.1038/s41467-023-37950-7 37296108 PMC10256802

[21] He Y-W, Deng Y, Miao Y, Chatterjee S, Tran TM, Tian J, Lindow S. 2023. DSF-family quorum sensing signal-mediated intraspecies, interspecies, and inter-kingdom communication. Trends Microbiol 31:36–50. doi:10.1016/j.tim.2022.07.006 35941062

[22] Rosales-Hurtado M, Meffre P, Szurmant H, Benfodda Z. 2020. Synthesis of histidine kinase inhibitors and their biological properties. Med Res Rev 40:1440–1495. doi:10.1002/med.21651 31802520 PMC7272307

[23] Lin L, Xu K, Shen D, Chou S-H, Gomelsky M, Qian G. 2021. Antifungal weapons of Lysobacter, a mighty biocontrol agent. Environ Microbiol 23:5704–5715. doi:10.1111/1462-2920.15674 34288318

[24] Yue H, Jiang J, Taylor AJ, Leite ADL, Dodds ED, Du L. 2021. Outer membrane vesicle-mediated codelivery of the antifungal HSAF metabolites and lytic polysaccharide monooxygenase in the predatory Lysobacter enzymogenes. ACS Chem Biol 16:1079–1089. doi:10.1021/acschembio.1c00260 34032403 PMC8797504

[25] Li Y, Wang H, Liu Y, Jiao Y, Li S, Shen Y, Du L. 2018. Biosynthesis of the polycyclic system in the antifungal HSAF and analogues from Lysobacter enzymogenes. Angew Chem Int Ed 57:6221–6225. doi:10.1002/anie.201802488 PMC6017996 29573092

[26] Lou L, Qian G, Xie Y, Hang J, Chen H, Zaleta-Rivera K, Li Y, Shen Y, Dussault PH, Liu F, Du L. 2011. Biosynthesis of HSAF, a tetramic acid-containing macrolactam from Lysobacter enzymogenes. J Am Chem Soc 133:643–645. doi:10.1021/ja105732c 21171605 PMC3078565

[27] Li Y, Chen H, Ding Y, Xie Y, Wang H, Cerny RL, Shen Y, Du L. 2014. Iterative assembly of two separate polyketide chains by the same single-module bacterial polyketide synthase in the biosynthesis of HSAF. Angew Chem Int Ed 53:7524–7530. doi:10.1002/anie.201403500 PMC4107061 24890524

[28] Xu K, Lin L, Shen D, Chou S-H, Qian G. 2021. Clp is a “busy” transcription factor in the bacterial warrior, Lysobacter enzymogenes. Comput Struct Biotechnol J 19:3564–3572. doi:10.1016/j.csbj.2021.06.020 34257836 PMC8246147

[29] Xu G, Han S, Huo C, Chin K-H, Chou S-H, Gomelsky M, Qian G, Liu F. 2018. Signaling specificity in the c-di-GMP-dependent network regulating antibiotic synthesis in Lysobacter. Nucleic Acids Res 46:9276–9288. doi:10.1093/nar/gky803 30202891 PMC6182147

[30] Chen Y, Xia J, Su Z, Xu G, Gomelsky M, Qian G, Liu F. 2017. Lysobacter PilR, the regulator of type IV pilus synthesis, controls antifungal antibiotic production via a cyclic di-GMP pathway. Appl Environ Microbiol 83. doi:10.1128/AEM.03397-16 PMC5359478 28087536

[31] Lin L, Yang Z, Tao M, Shen D, Cui C, Wang P, Wang L, Jing M, Qian G, Shao X. 2023. Lysobacter enzymogenes prevents Phytophthora infection by inhibiting pathogen growth and eliciting plant immune responses. Front Plant Sci 14:1116147. doi:10.3389/fpls.2023.1116147 36743479 PMC9892905

[32] Li X, Wang H, Li Y, Du L. 2019. Construction of a hybrid gene cluster to reveal coupled ring formation–hydroxylation in the biosynthesis of HSAF and analogues from Lysobacter enzymogenes. Med Chem Commun 10:907–912. doi:10.1039/C9MD00154A PMC6595964 31303988

[33] Yang M, Ren S, Shen D, Yang N, Wang B, Han S, Shen X, Chou S-H, Qian G. 2020. An intrinsic mechanism for coordinated production of the contact-dependent and contact-independent weapon systems in a soil bacterium. PLoS Pathog 16:e1008967. doi:10.1371/journal.ppat.1008967 33035267 PMC7577485

[34] Lu Z, Evans S, McDonnell L, Bandari NC, Weng Y, Jin W, Speight R, Schenk G, Howard CB, Vickers CE, Peng B. 2025. Exploiting the geranylgeranyl-pyrophosphate-sensing N-terminal domain of HMG-CoA reductase 2 to regulate farnesyl pyrophosphate synthase (Erg20p) for improved monoterpene production in Saccharomyces cerevisiae. Yeast 42:169–180. doi:10.1002/yea.4005 40686049

[35] Sudbery PE. 2011. Growth of Candida albicans hyphae. Nat Rev Microbiol 9:737–748. doi:10.1038/nrmicro2636 21844880

[36] Ramage G, Saville SP, Wickes BL, López-Ribot JL. 2002. Inhibition of Candida albicans biofilm formation by farnesol, a quorum-sensing molecule. Appl Environ Microbiol 68:5459–5463. doi:10.1128/AEM.68.11.5459-5463.2002 12406738 PMC129887

[37] Bröker JN, Müller B, van Deenen N, Prüfer D, Schulze Gronover C. 2018. Upregulating the mevalonate pathway and repressing sterol synthesis in Saccharomyces cerevisiae enhances the production of triterpenes. Appl Microbiol Biotechnol 102:6923–6934. doi:10.1007/s00253-018-9154-7 29948122 PMC6096838

[38] Florio AR, Ferrari S, De Carolis E, Torelli R, Fadda G, Sanguinetti M, Sanglard D, Posteraro B. 2011. Genome-wide expression profiling of the response to short-term exposure to fluconazole in Cryptococcus neoformans serotype A. BMC Microbiol 11:97. doi:10.1186/1471-2180-11-97 21569340 PMC3119188

[39] Su H, Han L, Ding N, Guan P, Hu C, Huang X. 2018. Bafilomycin C1 exert antifungal effect through disturbing sterol biosynthesis in Candida albicans. J Antibiot (Tokyo) 71:467–476. doi:10.1038/s41429-017-0009-8 29391532

[40] Sharma M, Prasad R. 2011. The quorum-sensing molecule farnesol is a modulator of drug efflux mediated by ABC multidrug transporters and synergizes with drugs in Candida albicans. Antimicrob Agents Chemother 55:4834–4843. doi:10.1128/AAC.00344-11 21768514 PMC3186959

[41] Kischkel B, Souza GK, Chiavelli LUR, Pomini AM, Svidzinski TIE, Negri M. 2020. The ability of farnesol to prevent adhesion and disrupt Fusarium keratoplasticum biofilm. Appl Microbiol Biotechnol 104:377–389. doi:10.1007/s00253-019-10233-2 31768611

[42] Zhu M, Li Y, Nan F, Wang J, Gao B, Song H, Bian Z, Wang X, Zhu Y, Wang Y. 2025. Mechanism of Lysobacter enzymogenes resistance toward fungi induced by fungal-derived signal α-terpinene. Appl Environ Microbiol 91:e0147125. doi:10.1128/aem.01471-25 40990487 PMC12542728

[43] Jacob-Dubuisson F, Mechaly A, Betton J-M, Antoine R. 2018. Structural insights into the signalling mechanisms of two-component systems. Nat Rev Microbiol 16:585–593. doi:10.1038/s41579-018-0055-7 30008469

[44] Zhu Y, Dou Q, Du L, Wang Y. 2023. QseB/QseC: a two-component system globally regulating bacterial behaviors. Trends Microbiol 31:749–762. doi:10.1016/j.tim.2023.02.001 36849330

[45] Gao R, Bouillet S, Stock AM. 2019. Structural basis of response regulator function. Annu Rev Microbiol 73:175–197. doi:10.1146/annurev-micro-020518-115931 31100988

[46] Chakraborty S, Winardhi RS, Morgan LK, Yan J, Kenney LJ. 2017. Non-canonical activation of OmpR drives acid and osmotic stress responses in single bacterial cells. Nat Commun 8:1587. doi:10.1038/s41467-017-02030-0 29138484 PMC5686162

[47] Yuan J, Jin F, Glatter T, Sourjik V. 2017. Osmosensing by the bacterial PhoQ/PhoP two-component system. Proc Natl Acad Sci USA 114:E10792–E10798. doi:10.1073/pnas.1717272114 29183977 PMC5740661

[48] Yadavalli SS, Carey JN, Leibman RS, Chen AI, Stern AM, Roggiani M, Lippa AM, Goulian M. 2016. Antimicrobial peptides trigger a division block in Escherichia coli through stimulation of a signalling system. Nat Commun 7:12340. doi:10.1038/ncomms12340 27471053 PMC4974570

[49] Abramson J, Adler J, Dunger J, Evans R, Green T, Pritzel A, Ronneberger O, Willmore L, Ballard AJ, Bambrick J, et al.. 2024. Accurate structure prediction of biomolecular interactions with AlphaFold 3. Nature 630:493–500. doi:10.1038/s41586-024-07487-w 38718835 PMC11168924

[50] Eberhardt J, Santos-Martins D, Tillack AF, Forli S. 2021. AutoDock Vina 1.2.0: new docking methods, expanded force field, and python bindings. J Chem Inf Model 61:3891–3898. doi:10.1021/acs.jcim.1c00203 34278794 PMC10683950

[51] Su Z, Han S, Fu ZQ, Qian G, Liu F. 2018. Heat-stable antifungal factor (HSAF) biosynthesis in Lysobacter enzymogenes is controlled by the interplay of two transcription factors and a diffusible molecule. Appl Environ Microbiol 84. doi:10.1128/AEM.01754-17 PMC5772243 29101199

[52] Ponde NO, Lortal L, Ramage G, Naglik JR, Richardson JP. 2021. Candida albicans biofilms and polymicrobial interactions. Crit Rev Microbiol 47:91–111. doi:10.1080/1040841X.2020.1843400 33482069 PMC7903066

[53] Cugini C, Calfee MW, Farrow JMR, Morales DK, Pesci EC, Hogan DA. 2007. Farnesol, a common sesquiterpene, inhibits PQS production in Pseudomonas aeruginosa. Mol Microbiol 65:896–906. doi:10.1111/j.1365-2958.2007.05840.x 17640272

[54] McAlester G, O’Gara F, Morrissey JP. 2008. Signal-mediated interactions between Pseudomonas aeruginosa and Candida albicans. J Med Microbiol 57:563–569. doi:10.1099/jmm.0.47705-0 18436588

[55] Bamford CV, d’Mello A, Nobbs AH, Dutton LC, Vickerman MM, Jenkinson HF. 2009. Streptococcus gordonii modulates Candida albicans biofilm formation through intergeneric communication. Infect Immun 77:3696–3704. doi:10.1128/IAI.00438-09 19528215 PMC2737996

[56] Shirtliff ME, Peters BM, Jabra-Rizk MA. 2009. Cross-kingdom interactions: Candida albicans and bacteria. FEMS Microbiol Lett 299:1–8. doi:10.1111/j.1574-6968.2009.01668.x 19552706 PMC4406406

[57] García-Calderón CB, García-Quintanilla M, Casadesús J, Ramos-Morales F. 2005. Virulence attenuation in Salmonella enterica rcsC mutants with constitutive activation of the Rcs system. Microbiology (Reading) 151:579–588. doi:10.1099/mic.0.27520-0 15699206

[58] Wall E, Majdalani N, Gottesman S. 2018. The complex Rcs regulatory cascade. Annu Rev Microbiol 72:111–139. doi:10.1146/annurev-micro-090817-062640 29897834

[59] Chen H, Fujita M, Feng Q, Clardy J, Fink GR. 2004. Tyrosol is a quorum-sensing molecule in Candida albicans. Proc Natl Acad Sci USA 101:5048–5052. doi:10.1073/pnas.0401416101 15051880 PMC387371

[60] Kovács R, Majoros L. 2020. Fungal quorum-sensing molecules: a review of their antifungal effect against Candida biofilms. J Fungi (Basel) 6:99. doi:10.3390/jof6030099 32630687 PMC7559060

[61] Perry EK, Meirelles LA, Newman DK. 2022. From the soil to the clinic: the impact of microbial secondary metabolites on antibiotic tolerance and resistance. Nat Rev Microbiol 20:129–142. doi:10.1038/s41579-021-00620-w 34531577 PMC8857043

[62] Zhu Y, Han Y, Liu G, Bian Z, Yan X, Li Y, Long H, Yu G, Wang Y. 2022. Novel indole-mediated potassium ion import system confers a survival advantage to the Xanthomonadaceae family. ISME J 16:1717–1729. doi:10.1038/s41396-022-01219-6 35319020 PMC9213462

[63] Gregus P, Vlcková H, Buchta V, Kestranek J, Krivcíková L, Nováková L. 2010. Ultra high performance liquid chromatography tandem mass spectrometry analysis of quorum-sensing molecules of Candida albicans. J Pharm Biomed Anal 53:674–681. doi:10.1016/j.jpba.2010.05.029 20580513

[64] Tang B, Sun C, Zhao Y, Xu H, Xu G, Liu F. 2018. Efficient production of heat-stable antifungal factor through integrating statistical optimization with a two-stage temperature control strategy in Lysobacter enzymogenes OH11. BMC Biotechnol 18:69. doi:10.1186/s12896-018-0478-2 30355310 PMC6201579

[65] Zhong J, Yan X, Zuo X, Zhao X, Yang J, Dou Q, Peng L, Zhu Y, Xiao Y, Bian Z, He D, Xu Q, Wright S, Li Y, Du L, Wang Y, Yuan J. 2022. Developing a new treatment for superficial fungal infection using antifungal Collagen-HSAF dressing. Bioeng Transl Med 7:e10304. doi:10.1002/btm2.10304 36176602 PMC9472023

[66] Jian L, Nan F, Zhang Y, Jiang Y, Sun M, Yang W, Zhu Y, Zhang Q, Li Y, Yan X, Yuan X, Han L, Wang Y. 2026. Dual-targeting nanotherapy disrupts fungal-bacterial synergy to reprogram inflammatory microenvironments in periodontitis. Biomaterials 329:123923. doi:10.1016/j.biomaterials.2025.123923 41420961

[67] Song H, Zhu Y, Qu Z, Zhu M, Li X, Zhao L, Wang K, Zhang R, Cui L, Li Y, Bian Z, Zhang W, Chen Y, Du L, Wang J-L, Zhao X, Deng L, Wang Y. 2025. Two-step localization driven by peptidoglycan hydrolase in interbacterial predation. ISME J 19:wraf208. doi:10.1093/ismejo/wraf208 40972874 PMC12510463

[68] Chen J, Zhou Y, Liu Z, Lu Y, Jiang Y, Cao K, Zhou N, Wang D, Zhang C, Zhou N, Shi K, Zhang L, Zhou L, Wang Z, Zhang H, Tang K, Ma J, Lv J, Huang B. 2024. Hepatic glycogenesis antagonizes lipogenesis by blocking S1P via UDPG. Science 383:eadi3332. doi:10.1126/science.adi3332 38359126

[69] Bothe S, Hänzelmann P, Böhler S, Kehrein J, Zehe M, Wiedemann C, Hellmich UA, Brenk R, Schindelin H, Sotriffer C. 2022. Fragment screening using biolayer interferometry reveals ligands targeting the SHP-motif binding site of the AAA+ ATPase p97. Commun Chem 5:169. doi:10.1038/s42004-022-00782-5 36697690 PMC9814400

[70] Fan R, Li Z, Shi X, Wang L, Zhang X, Dong Y, Quan C. 2022. Expression, purification, and characterization of the recombinant, two-component, response regulator ArlR from Fusobacterium nucleatum. Appl Biochem Biotechnol 194:2093–2107. doi:10.1007/s12010-021-03785-5 35029789

[71] Ji D, Mohsen MG, Harcourt EM, Kool ET. 2016. ATP-releasing nucleotides: linking DNA synthesis to luciferase signaling. Angew Chem Int Ed Engl 55:2087–2091. doi:10.1002/anie.201509131 26836342 PMC4955596

[72] Liu B, Liu Y, Yang B, Wang Q, Liu X, Qin J, Zhao K, Li F, Feng X, Li L, Wu P, Liu M, Zhu S, Feng L, Wang L. 2022. Escherichia coli O157:H7 senses microbiota-produced riboflavin to increase its virulence in the gut. Proc Natl Acad Sci USA 119:e2212436119. doi:10.1073/pnas.2212436119 36409903 PMC9860305

[73] Letunic I, Khedkar S, Bork P. 2021. SMART: recent updates, new developments and status in 2020. Nucleic Acids Res 49:D458–D460. doi:10.1093/nar/gkaa937 33104802 PMC7778883

[74] Hallgren J, Tsirigos KD, Pedersen MD, Almagro Armenteros JJ, Marcatili P, Nielsen H, Krogh A, Winther O. 2022. DeepTMHMM predicts alpha and beta transmembrane proteins using deep neural networks. bioRxiv. doi:10.1101/2022.04.08.487609

