# Supplementary material for: Hijacking competitor-derived signals: RcsB/C drives Lysobacter enzymogenes to exploit farnesol for enhanced antifungal capacity
Source: Appl Environ Microbiol. 2026 Apr 3;92(4):e00304-26. doi: 10.1128/aem.00304-26 (PMC13101495; doi:10.1128/aem.00304-26)
Supplement: Supplemental material — Fig. S1 to S17; Tables S1 to S3. [file aem.00304-26-s0001.pdf]

Supplementary material

Hijacking competitor-derived signals: RcsB/C drives *Lysobacter enzymogenes* to exploit farnesol for enhanced antifungal capacity

Supplementary figures

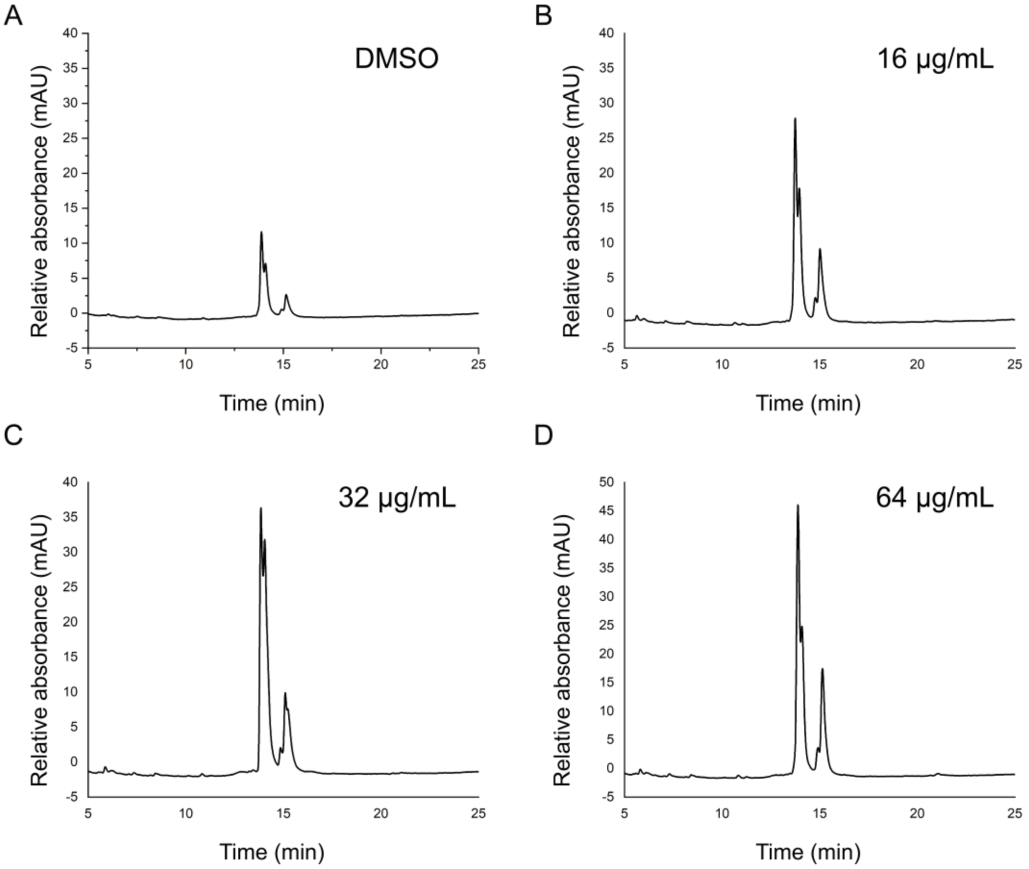

Fig. S1 The effect of different concentrations of *C. krusei* extract on the biosynthesis of *Le*YC36 HSAF. (A) is the control, and **(B), (C)** and **(D)** are 16, 32, and 64 μg/mL, respectively.

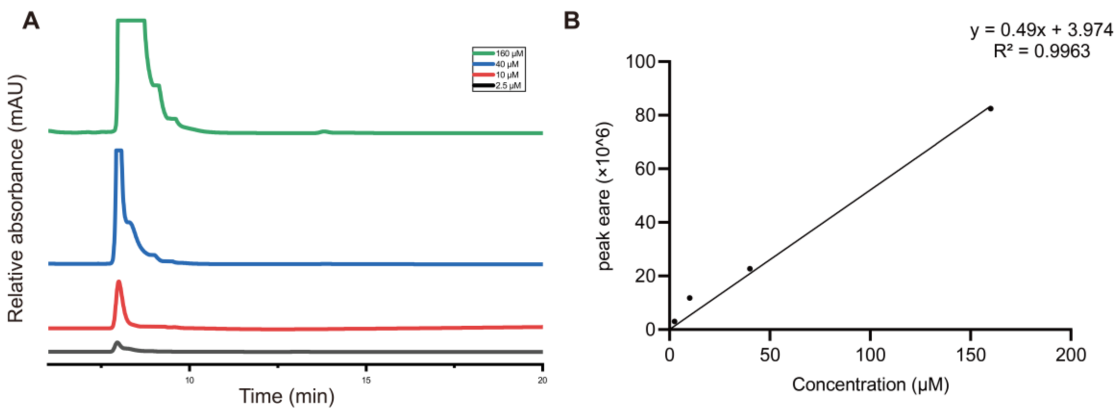

Fig. S2 Peak area-concentration chromatogram and corresponding standard curve of farnesol standard. (A) Chromatogram of farnesol standard. **(B)** Standard curve of farnesol standard (plot of peak area vs. concentration).

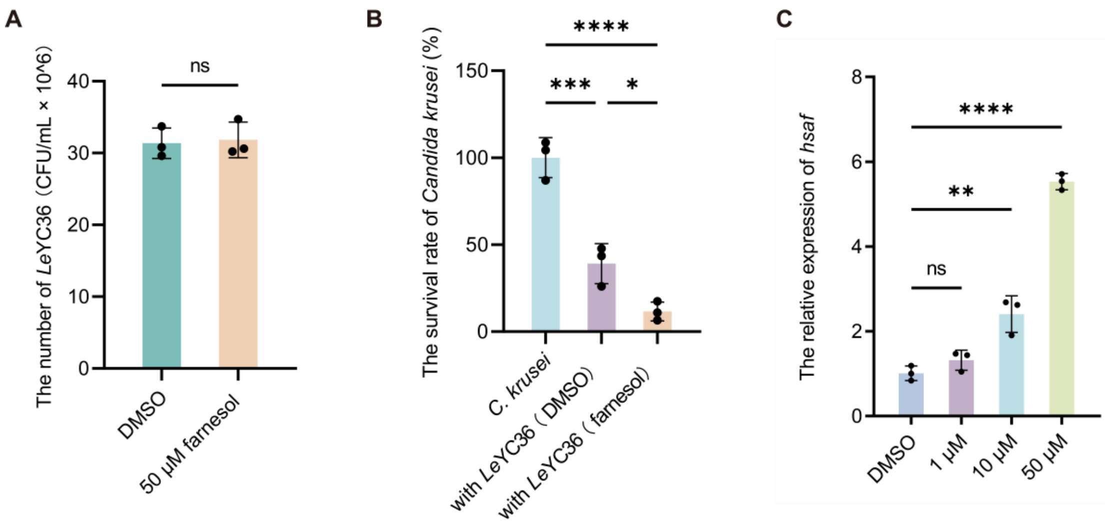

Fig. S3 Effects of farnesol on survival rate and antifungal activity of *Le*YC36. (A) Effect of 50 μM farnesol on the survival rate of *Le*YC36. **(B)** Effect of exogenous addition of 50 μM farnesol on the survival rate of *Candida krusei*. **(C)** Effects of different concentrations of farnesol on the expression levels of key HSAF biosynthetic genes in *Le*YC36 after 12 hours. ns, not significant, **P* value < 0.05, ***P* value < 0.01, ****P* value < 0.001, *****P* value < 0.0001. Error bars indicate the Standard Deviation (SD) of three replicates. Data presented as mean ± SD.

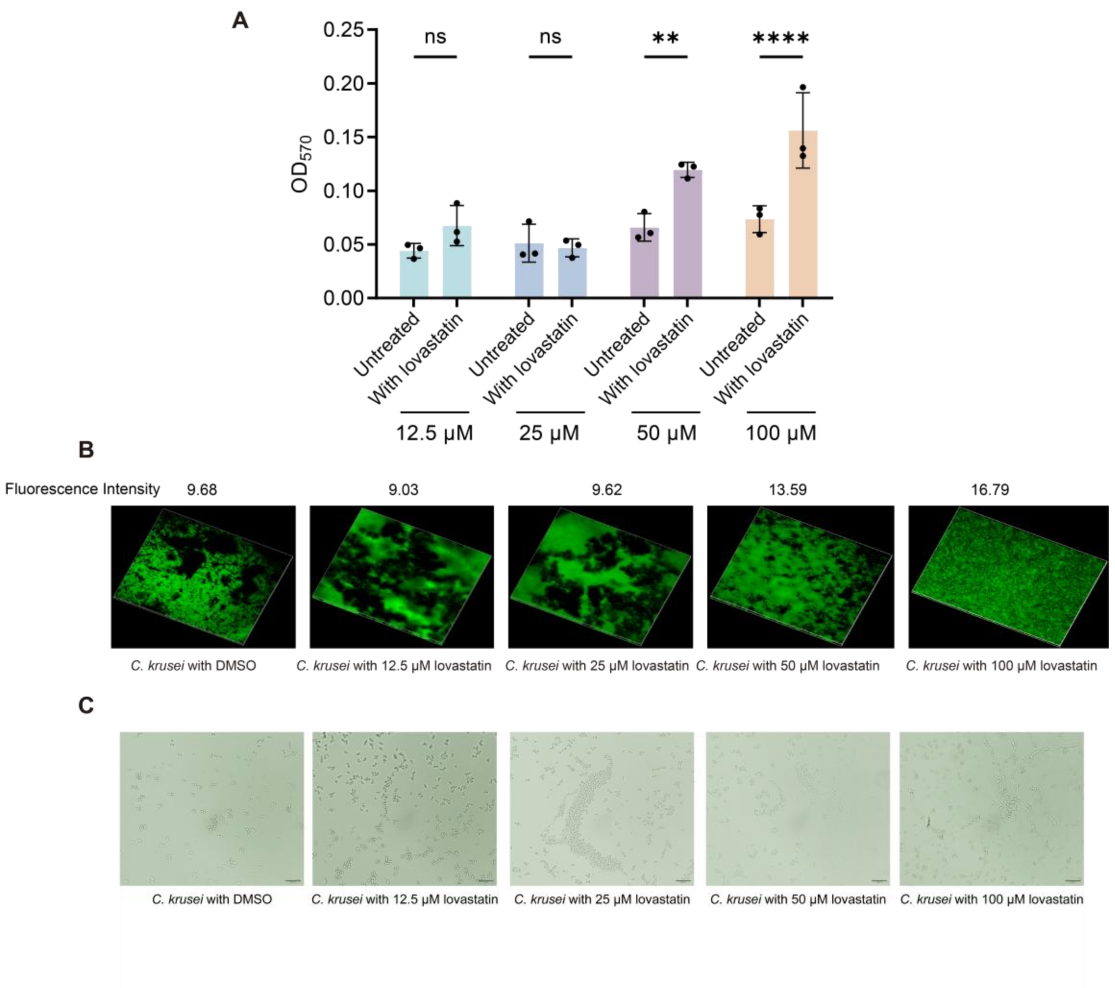

Fig. S4 Effects of different concentrations of lovastatin on biofilm formation and cell morphology of *Candida krusei*. (A) Effects of different concentrations of lovastatin on biofilm formation of *C. krusei*. **(B)** Visualization of biofilm alterations under various treatment conditions using confocal laser scanning microscopy. **(C)** Effects of different concentrations of lovastatin on the cell morphology of *C. krusei*. ns, not significant, ***P* value < 0.01, *****P* value < 0.0001. Error bars indicate the SD of three replicates. Data presented as mean ± SD.

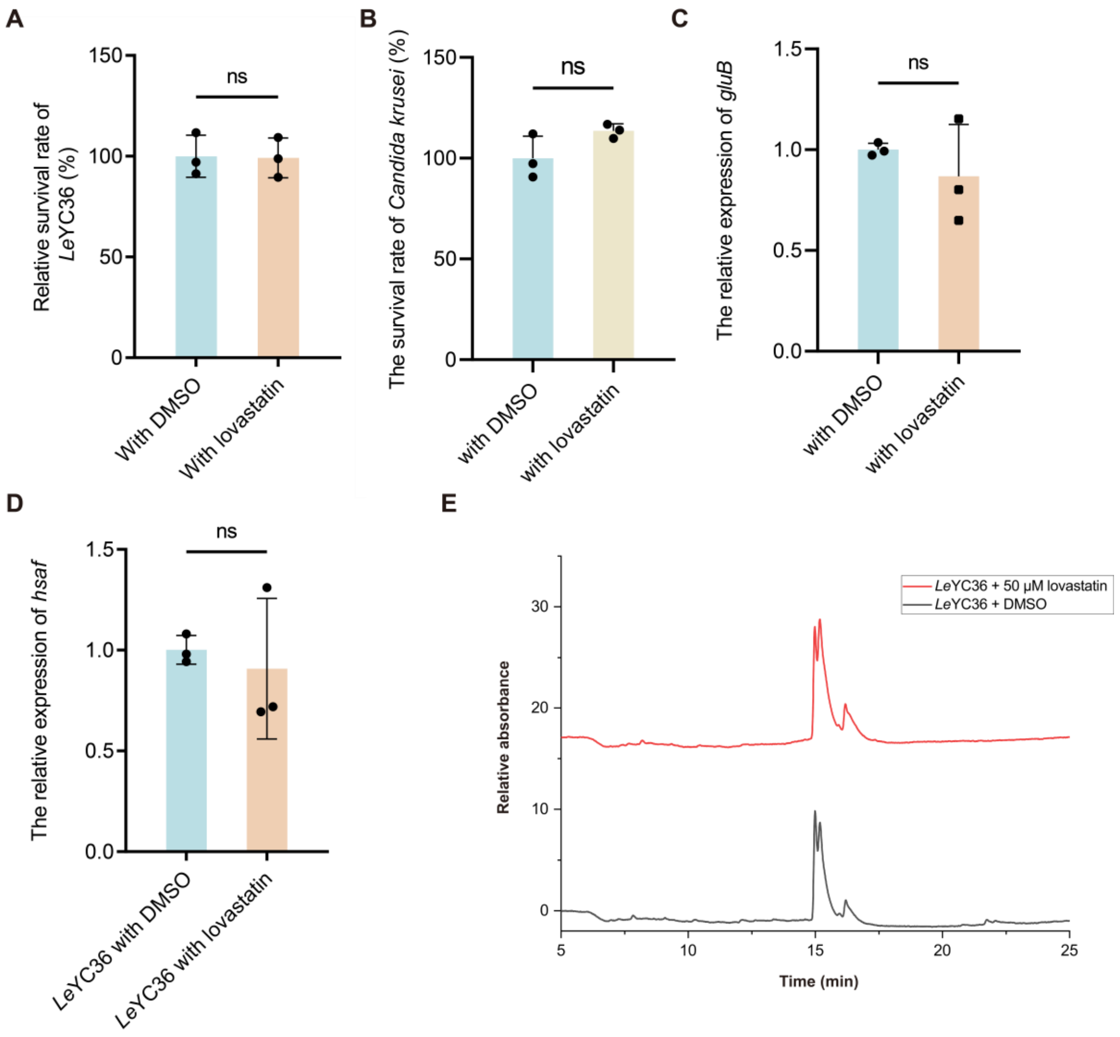

Fig. S5 The effect of lovastatin on *Le*YC36. (A) The effect of lovastatin on *Le*YC36 biomass. **(B)** The effect of lovastatin on the survival rate of *Candida krusei*. **(C)** The effect of lovastatin on the expression of the *gluB* gene. **(D, E)** The effect of lovastatin on *Le*YC36 HSAF expression and synthesis levels as detected by real-time PCR and HPLC. Error bars indicate the SD of three replicates. Data presented as mean ± SD. For significance information, ns, not significant.

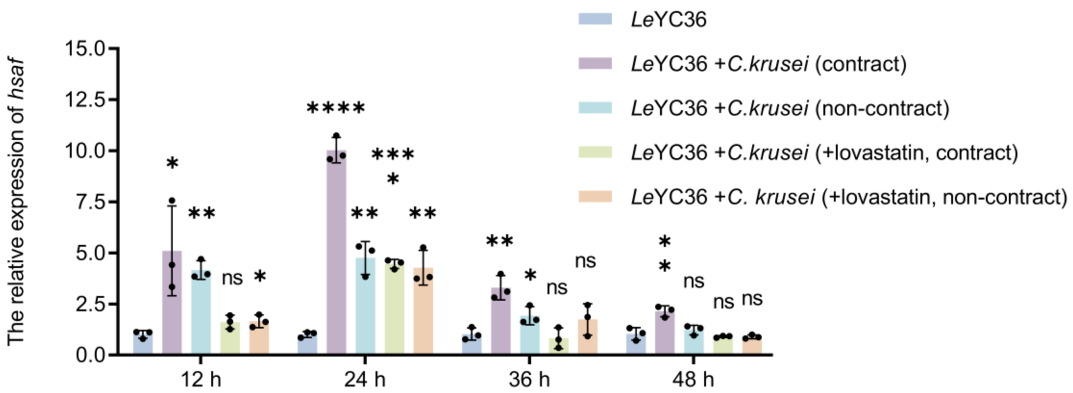

Fig. S6 Effect of lovastatin addition on HSAF gene expression in *L. enzymogenes* in co-culture systems. ns, not significant, **P* value < 0.05, ***P* value < 0.01, *****P* value < 0.0001. Error bars indicate the SD of three replicates. Data presented as mean ± SD.

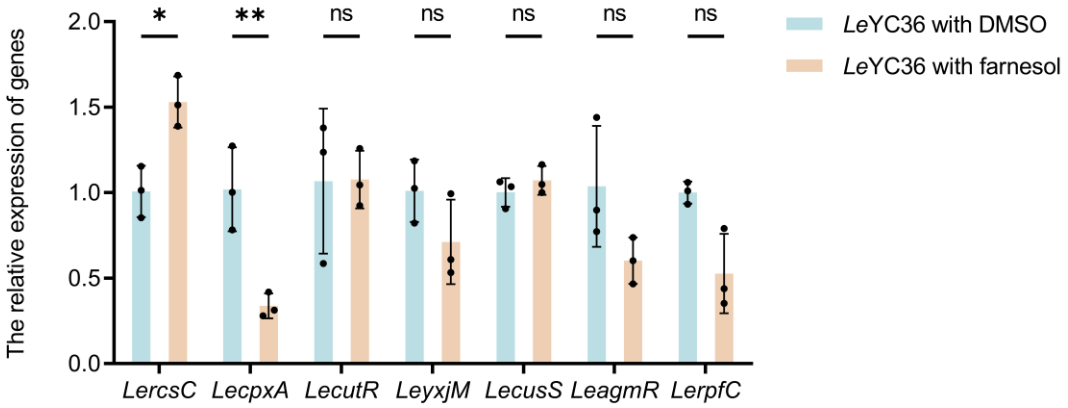

Fig. S7 Real-time PCR identifies potential TCSs. Error bars indicate the SD of three replicates. Data presented as mean ± SD. For significance information, ns, not significant, **P* value < 0.05, ***P* value < 0.01.

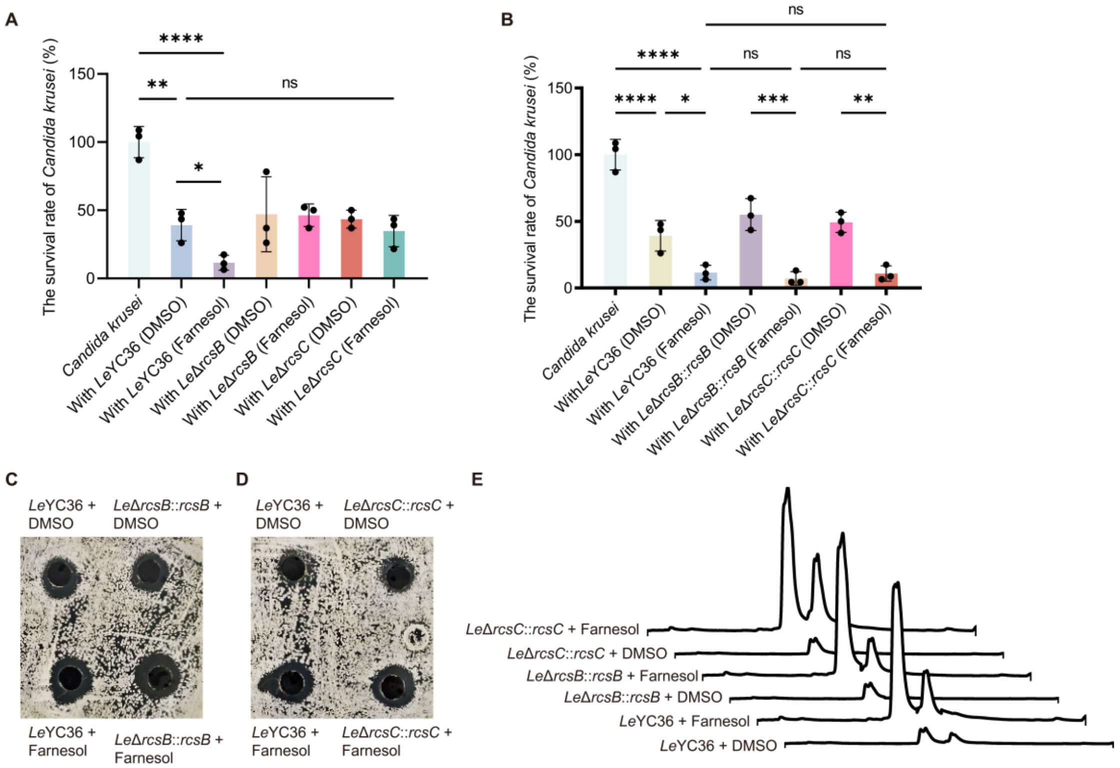

Fig. S8 Effects of two-component system gene deletion and complementation on the physiology of *Le*YC36. (A) Effects of *Le*Δ*rcsB* and *Le*Δ*rcsC* on the survival rate of *C. krusei*. **(B)** Effects of *Le*Δ*rcsB*::*rcsB* and *Le*Δ*rcsC*::*rcsC* on the survival rate of *C. krusei***.** **(C)** Antifungal ability of *Le*Δ*rcsB*::*rcsB* against fungi following exogenous farnesol addition. **(D)** Antifungal ability of *Le*Δ*rcsC*::*rcsC* against fungi following exogenous farnesol addition. **(E)** HSAF production in *Le*Δ*rcsB::rcsB* and *Le*Δ*rcsC::rcsC* following exogenous farnesol addition. Error bars indicate the SD of three replicates. Data presented as mean ± SD. For significance information, ns, not significant, ***P* value < 0.01, ****P* value < 0.001, ****P* value < 0.0001.

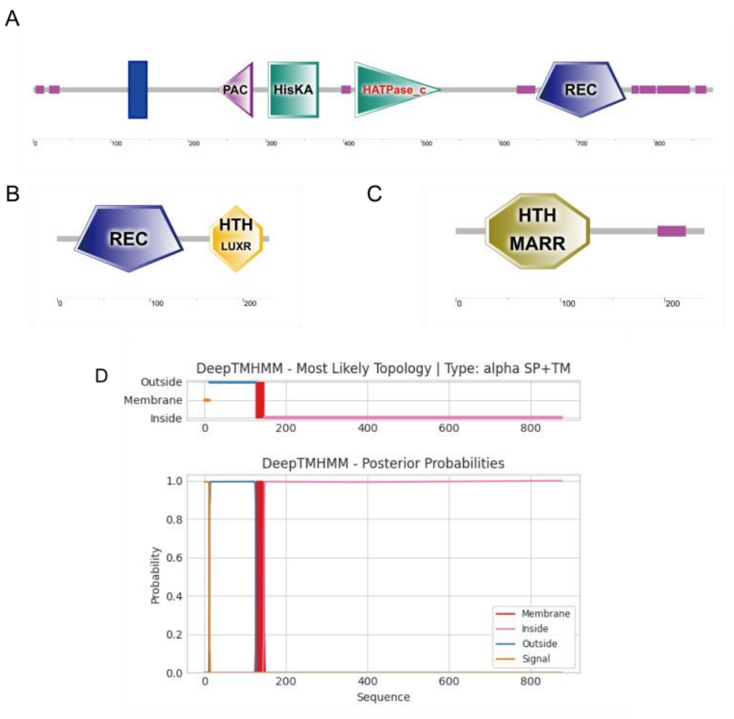

Fig. S9 Protein domains of RcsC, RcsB and MarR-2 predicted with online analysis. **(A)** Predicted domains of RcsC. RcsC contains Motif C-terminal to PAS motifs domain, His Kinase A (phosphoacceptor) domain, Histidine kinase-like ATPases domain, and REC domain. **(B)** Predicted domains of RcsB. RcsB contains REC domain and HTH-LUXR domain. **(C)** Predicted domains of MarR-2. MarR-2 contains HTH-MARR domain. **(D)** Predicted transmembrane regions of RcsC.

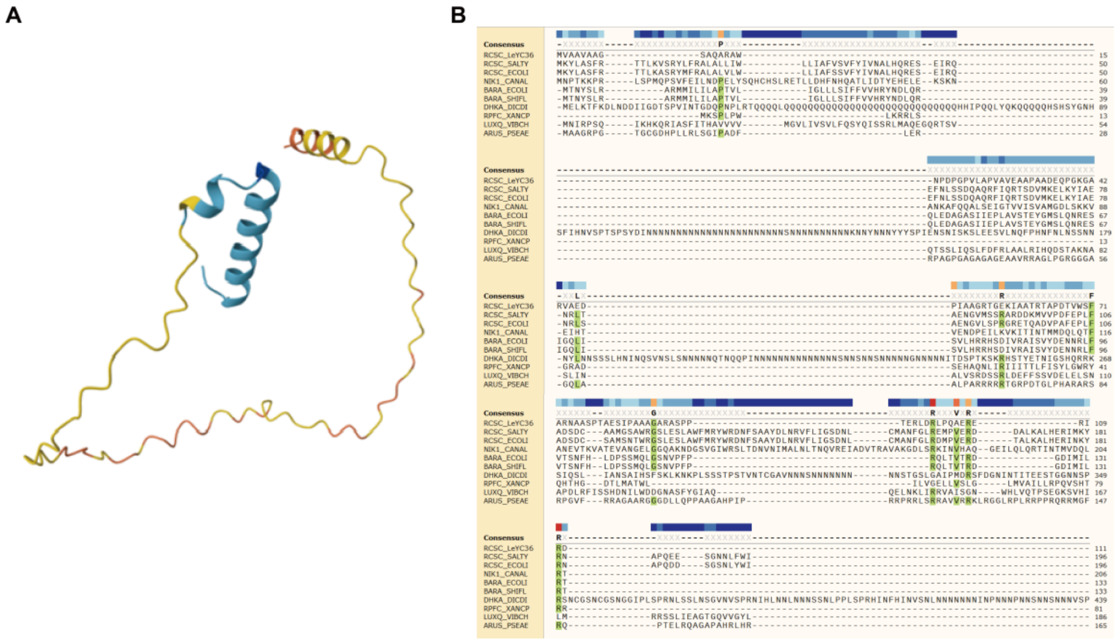

Figure S10. Structural prediction of RcsC$_{PD}$. (A) Predicted structure of RcsC$_{PD}$ generated by AlphaFold3. **(B)** Conserved site analysis of RcsC$_{PD}$.

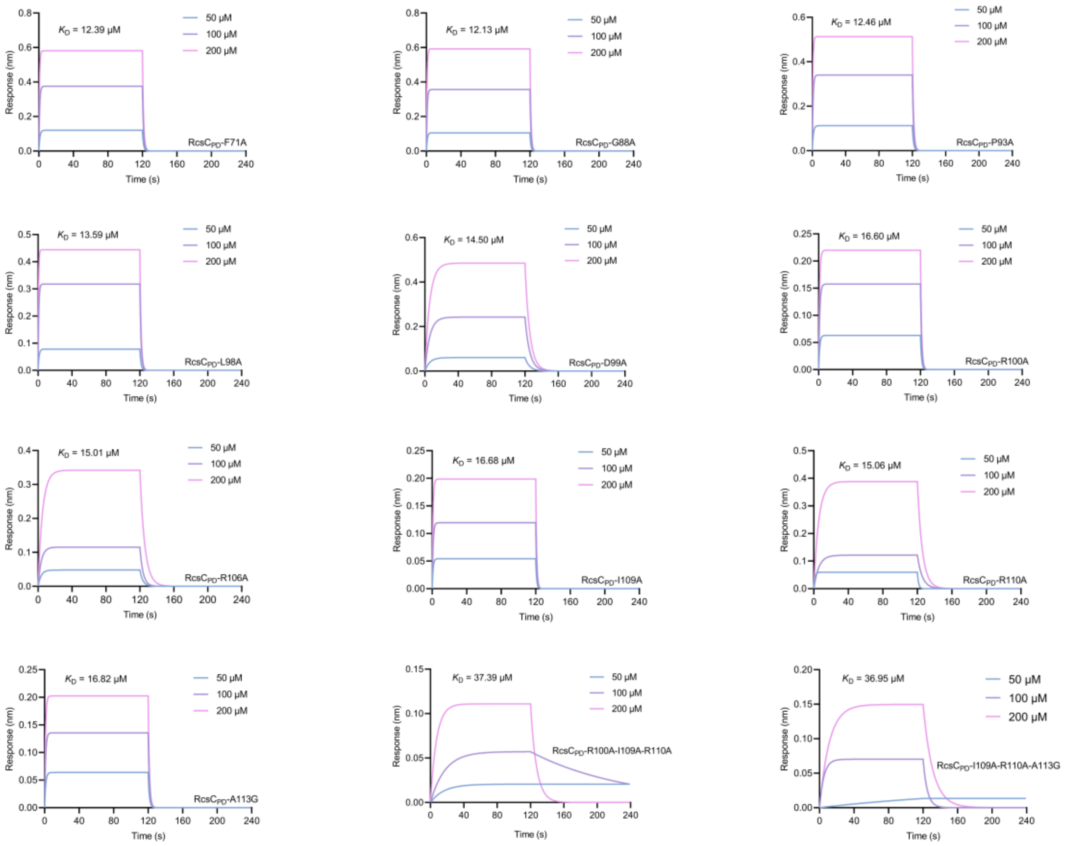

Fig. S11 BLI curves for the binding of different mutant proteins to farnesol. (A–J) represent the binding capacity of different RcsC mutant proteins (F71A, G88A, P93A, L98A, D99A, R100A, R106A, I109A, R110A, A113G, R100A-I109A-R110A and I109A-R110A-A113G) to farnesol.

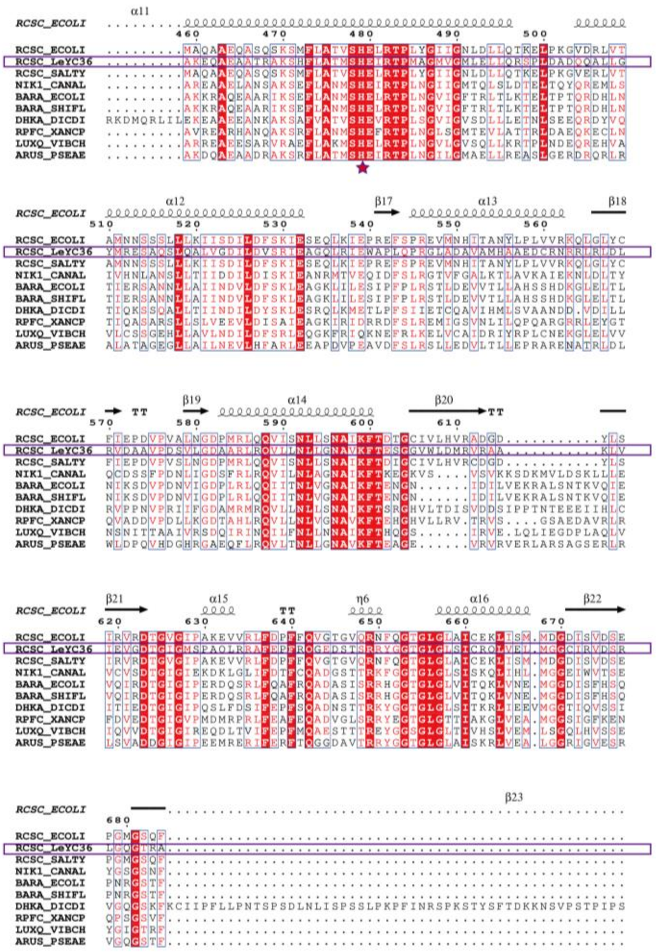

Fig. S12 The protein sequence alignment of RcsC from different bacterial species. Red parts represent the completely conserved sites. Due to the length of the sequence, this result only displays the sequence near the conserved His site. Results displayed by ESPript 3.0[1]. The red five-pointed star represent potential conserved phosphorylation sites, including the reported phosphorylation site of RstC (P0DMC5), as well as possible phosphorylation sites of *Le*RcsC and other RstC proteins. The identity among amino acid positions is highlighted in red, with dark red and light red indicating high and low identity of amino acid positions, respectively.

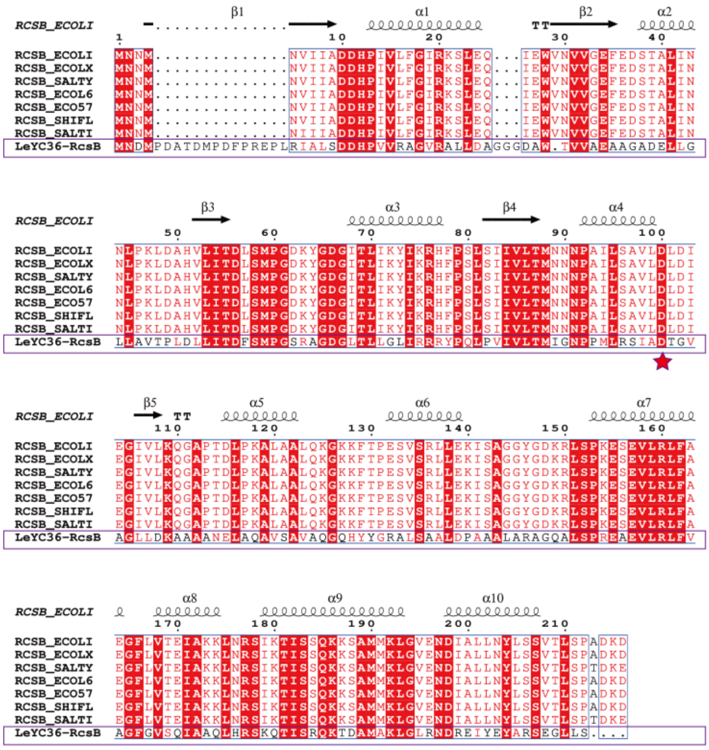

Fig. S13 The protein sequence alignment of RcsB from different bacterial species. Red parts represent the completely conserved sites. Results displayed by ESPript 3.0[1]. The red five-pointed star represent potential conserved phosphorylation sites, including the reported phosphorylation site of RstB (P0DMC7), as well as possible phosphorylation sites of *Le*RcsB and other RstB proteins. The identity between amino acid sites is indicated by red highlighting, where dark red and light red represent high and low identity of amino acid sites, respectively.

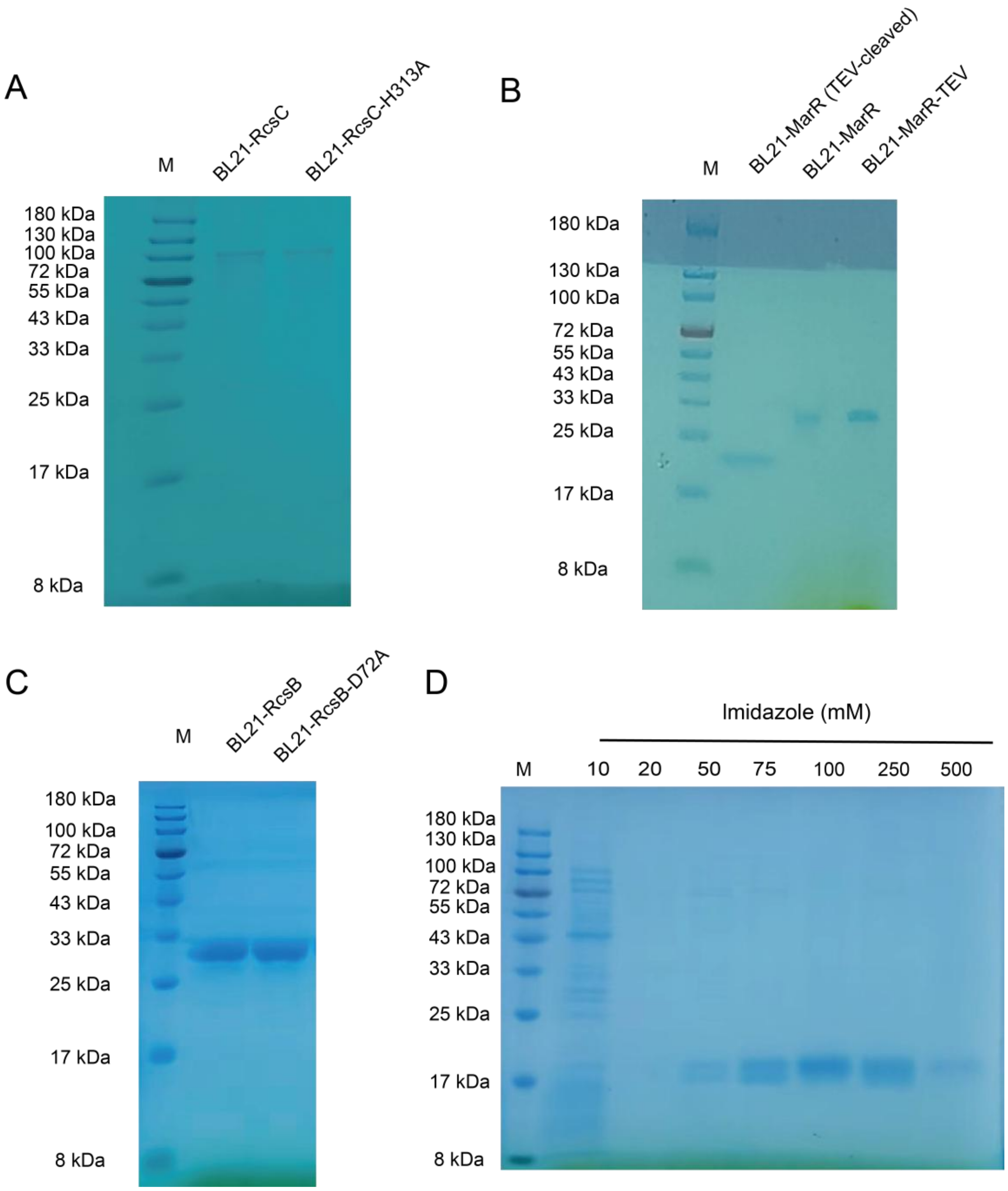

Fig. S14 SDS-PAGE assay for protein purifications. SDS-PAGE result shows the pureness of recombinant **(A)** RcsC, RcsC-H313A, **(B)** MarR-2 and MarR-2 (TEV-cleaved), **(C)** RcsB, RcsB-D72A, **(D)** $RcsC_{PD}$ and its site-directed mutant proteins. M refers to Marker.

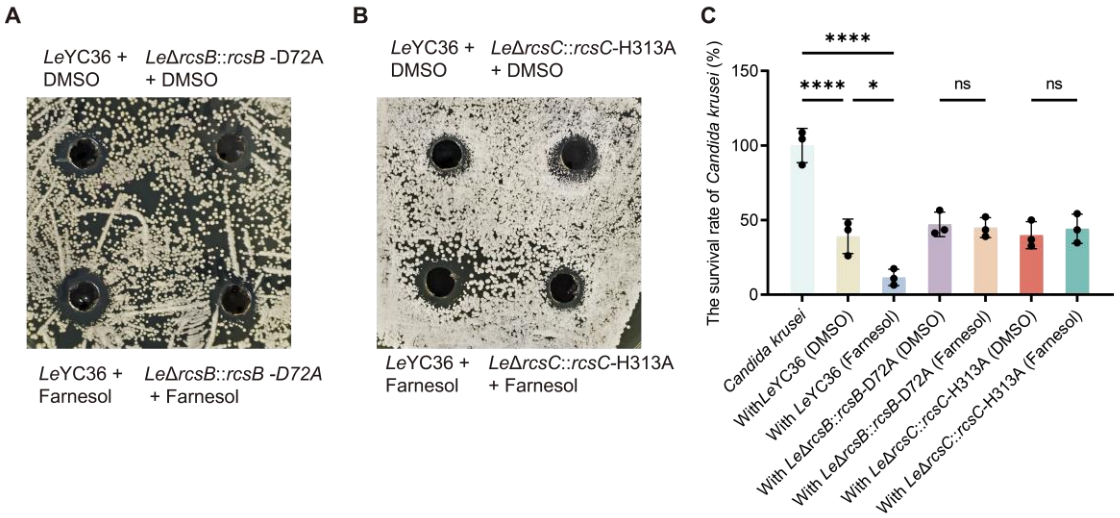

Fig. S15 Antifungal effects of *Le*Δ*rcsB*::*rcsB*-D72A and *Le*Δ*rcsC*::*rcsC*-H313A. (A) Antifungal activity of *Le*Δ*rcsB*::*rcsB*-D72A with or without farnesol treatment. **(B)** Antifungal activity of *Le*Δ*rcsC*::*rcsC*-H313A with or without farnesol treatment. **(C)** Effects of different strains on the survival rate of *Candida krusei* under conditions with or without farnesol treatment. Error bars indicate the SD of three replicates. Data presented as mean ± SD. For significance information, ns, not significant, *P value < 0.05, ****P value < 0.0001.

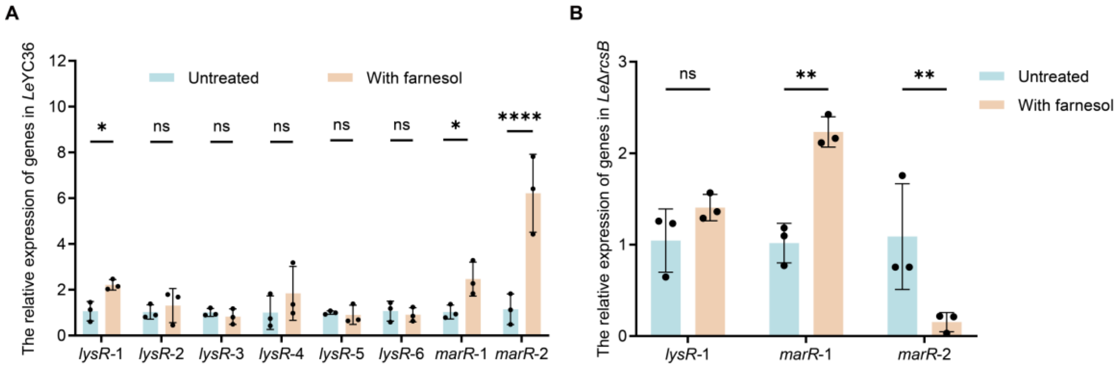

Fig. S16 Relative expression levels of *lysR* and *marR* genes. (A) Relative expression levels of potential downstream genes in wild-type *Le*YC36 with or without farnesol treatment. **(B)** Relative expression levels of potential downstream genes in *Le*Δ*rcsB* with or without farnesol treatment. Error bars indicate the SD of three replicates. Data presented as mean ± SD. For significance information, ns, not significant, **P* value < 0.05, ***P* value < 0.01, *****P* value < 0.0001.

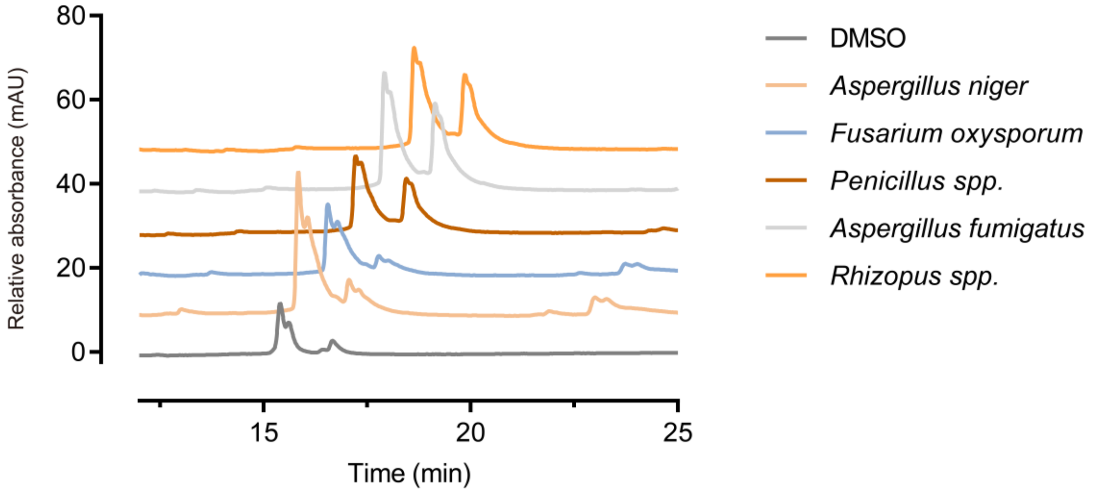

Fig. S17 The effect of different fungal secretions on the amount of *Le*YC36 HSAF synthesized.

Supplementary Tables

**Strains**	**Relevant characteristics**	**Source**
L. enzymogenes YC36	Wild-type strain	Lab storage
Candida krusei	Wild-type strain	Lab storage
E. coli BL21 (DE3)	*E. coli* strain for protein expression	Lab storage
E. coli S17-1 λpir	*E. coli* strain used as bacterial conjugal donor	Lab storage
*Le*Δ*hsaf*	*hsaf*-deletion *Le*YC36 mutant strain	Lab storage
*Le*Δ*rcsC*	*rcsC*-deletion *Le*YC36 mutant strain	This study
*Le*Δ*rcsB*	*rcsB*-deletion *Le*YC36 mutant strain	This study
*Le*Δ*marR-2*	*marR-2*-deletion *Le*YC36 mutant strain	This study
*Le*Δ*lysR-1*	*lysR-1*-deletion *Le*YC36 mutant strain	This study
RcsC-BL21	*E. coli* BL21 (DE3) strain for recombinant RcsC purification	This study
RcsB-BL21	*E. coli* BL21 (DE3) strain for recombinant RcsB purification	This study
RcsC-H313A-BL21	*E. coli* BL21 (DE3) strain for recombinant RcsC-H313A purification	This study
RcsC$_{PD}$-BL21	*E. coli* BL21 (DE3) strain for recombinant RcsC$_{PD}$ purification	This study
RcsB-D72A-BL21	*E. coli* BL21 (DE3) strain for recombinant RcsB-D72A purification	This study
MarR-2-BL21	*E. coli* BL21 (DE3) strain for recombinant MarR-2 purification	This study
**Plasmids**	**Relevant characteristics**	**Source**
pEX18Gm-*rcsC*	The vector, carrying upstream and downstream sequences of *rcsC*, used for *rcsC* knock-out	This study
pEX18Gm-*rcsB*	The vector, carrying upstream and downstream sequences of *rcsB*, used for *rcsB* knock-out	This study
pEX18Gm-*marR-2*	The vector, carrying upstream and downstream sequences of *marR-2*, used for *marR-2* knock-out	This study
pEX18Gm-*lysR-1*	The vector, carrying upstream and downstream sequences of *lysR-1*, used for *lysR-1* knock-out	This study
RcsC-pET-19b	The vector for recombinant RcsC expression	This study
RcsC-H313A-pET-19b	The vector for recombinant RcsC -H313A expression	This study
RcsB-pET-28a (+)	The vector for recombinant RcsB expression	This study
RcsB-D72A-pET-28a (+)	The vector for recombinant RcsB-D72A expression	This study
RcsC$_{PD}$-pET-28a (+)	The vector for recombinant RcsC$_{PD}$ expression	This study
MarR-2-pET-28a (+)	The vector for recombinant MarR-2 expression	This study
MarR-2-TEV-pET-28a (+)	The vector for recombinant MarR-2-TEV expression	This study

Table S1 All bacterial strains and plasmids used in this study. Including laboratory storage and obtained in this study.

Primers	Sequence	Purpose
rcsC-U-F- EcoR I	ccggaattctcggcaacccgccga	For gene in frame deletion
rcsC-U-R	tcagttcgcggttgatcgtgtcgggcgcggt	
rcsC-D-F	accgcgcccgacacgatcaaccgcgaactga	
rcsC-D-R- Hind III	cccaagcttgcccgccactgaaac	
rcsB-U-F- EcoR I	ccggaattcagcaccctgaacgagt	
rcsB-U-R	acaaggcgcggccgtgtctgacaaggcgat	
rcsB-D-F	atcgccttgtcagacacggccgcgccttgt	
rcsB-D-R- Hind III	cccaagcttagcacccggctccac	
lysR-1-up-F	aattcgagctcggtacccggggatacatctcctccgacgcc	
lysR-1-up-R	acccatctcttcatccagccacgccgaaactgcgcagggcggt	
lysR-1-down-F	cccccctgaccgccctgcgcagtttcggcgtggctggatgaagagatg	
lysR-1-down-R	gtaaaacgacggccagtgccacgctgcctgcggcgttggcg	
marR-2-up-F	aattcgagctcggtacccgggggacgaaaccccgaccaacg	
marR-2-up-R	cgccggcgccggcgcggaaccgcgccttgtcctcgcccagcagct	
marR-2-down-F	gttggagctgctgggcgaggacaaggcgcggttccgcgccggcgc	
marR-2-down-R	gtaaaacgacggccagtgccacgatcccatcgcggtctcccc	
rcsC-NdeI-F	ggaattccatatgatggtggcggccgtggcggcgggtt	Recombinant protein purification and site directed mutation
rcsC-BamH I-R	cgcggatccctaggcctcggccagcgcttcccag	
rcsB-EcoR I-F	ccggaattcgtgaacgacatgcccg	
rcsB-Hind III-R	cccaagctttcaggacagcaggcct	
marR-2-BamH I-F	cgcggatccatgagcgatcccgctttc	
marR-2-EcoR I-R	ccggaattcctattccgcggctgcg	
marR-2-TEV-F	cgcggatccgaaaatctttatttccaaggtatgagcgatcccgctttc	
rcsC$_{PD}$-BamH I-F	cgcggatccatggtggcggccgtg	
rcsC$_{PD}$-EcoR I-R	ccggaattccggcacgccctggcc	
rcsC-H313A-F	tgagcgctgagatccgcacgccgatggc	
rcsC-H313A-R	tctcagcgctcatcgtcgccaggaaatg	
rcsB-D72A-F	gctgatcaccgccttttccatgcccggcag	
rcsB-D72A-R	atggaaaaggcggtgatcagcaaatccagcggcg	
rcsC-F71A-F	gtttggtcggccgcacgaaacgcggccagt	
rcsC-F71A-R	gtttcgtgcggccgaccaaaccgtgtcgg	
rcsC-G88A-F	gccgccgccgccgcccgtgcgagcccgcc	
rcsC-G88A-R	cacgggcggcggcggcggcggggatcgatt	
rcsC-R100A-F	gctcgacgccctgccgcaggccgagcg	
rcsC-R100A-R	tgcggcagcgggtcgagccgctcggtcggc	
rcsC-R106A-F	gccgaggccgaacgcatccgcgacggcgcgac	
rcsC-R106A-R	atgcgttcggcctcggcctgcggcaggcgg	
rcsC-R110A-F	cgcatcgccgacggcgcgaccgccgcgcgctat	

rcsC-R110A-R	gcgccgtccgggatgcgttcgcgctcgg	
rcsC-P93A-F	tgcgagcgcgccgaccgagcggctcgaccgcct	
rcsC-P93A-R	ggtcggcgcgctcgcacgggcgccggc	
rcsC-L98A-F	accgagcgggccgaccgcctgccgcaggccgagcg	
rcsC-L98A-R	ggcggtcggcccgctcggtcggcgggctcgca	
rcsC-D99A-F	gagcggctcgcccgcctgccgcaggccgagcg	
rcsC-D99A-R	ggcaggcgggcgagccgctcggtcggcgggc	
rcsC-I109A-F	agcgcgaacgcgcccgcgacggcgcgaccgccgcgc	
rcsC-I109A-R	cgccgtcgcgggcgcgttcgcgctcggcctgc	
rcsC-A113G-F	atccgcgacggcgggaccgccgcgcgctat	
rcsC-A113G-R	ggcggtcccgccgtcgcggatgcgttcgcgc	
16S-F	gacgtcatcgtcagcaatcc	For real-time PCR
16S-R	gttggcgaccatccagaaac	
hsaf-F	gtctcggcgatggtgtattg	
hsaf-R	gtcgaaccggatctcgtact	
gluB-F	ctcgacccgatctgctactc	
gluB-R	tgtagccggtcaggtagttc	
rcsC-F	gataccggcatcggcatgag	
rcsC-R	gatgcatccgcccatcaac	
rcsB-F	gccgctggatttgctgat	
rcsB-R	gatcgaacgcagcatcgg	
cpxA-F	cgaggcctacacctaccag	
cpxA-F	ccagaaacggtagaccagga	
cutR-F	gccgacgactatctggtcaa	
cutR-R	cagcagggcgaattccttc	
yxjM-F	ggtcaaggaacacgtgcag	
yxjM-R	cgatgtccatgcggatgtc	
cusS-F	ggtggtgatgaaccttctgc	
cusS-R	acggattgagccggtagaac	
agmR-F	ttgccggtggtgatcatttc	
agmR-R	ctgcggcgagaaccagac	
rpfC-F	tcgtggaaagcatcgaagtg	
rpfC-R	gtagcggttgttctgcgaat	
lysR-1-F	cgaccttgatctcggtggaa	
lysR-1-R	ggcatacatcgcgtaatgct	
lysR-2-F	gactgcatccatttccgctt	
lysR-2-R	gaccaggaaatccaccgagt	
lysR-3-F	gcagctgagcctgtcctc	
lysR-4-R	cggatacagcaaggcgatg	
lysR-5-F	cttgcggatcacgattccag	
lysR-5-R	cattcgacaccagcagatcg	
lysR-6-F	gatcgaagtgttgtcgccat	
lysR-6-R	gcaggaaatccacgaacacc	

marR-1-F	caaactctcgcggctgatg	
marR-1-R	ggatcgagtactggttgaggt	
marR-2-F	ttcgctgcagttgttgatcc	
marR-2-R	gtgttcggcgcaatccac	
EGR13-F	tcaaccagctttgcaagtcc	
EGR13-R	tgcagacacatcacctttgac	
ACT1-F	ggtgatgaagcccaatccaa	
ACT1-R	ttggagcttcggtcaacaaa	
HMG1-F	tacctgttggagttgctggt	
HMG1-R	gaagcgaacacatggacctc	
ERG12-F	ggattgataacgcagtggct	
ERG12-R	ctgccaacaccagcaactaa	
ERG8-F	ctcgtgccattaccgaagtg	
ERG8-R	gcagttgcaacatcaaaccc	
MVD-F	ggctttgcagcattggtttc	
MVD-R	cacggcctttgagtcttgtc	
$P_{Unrelated}$-F	aggacgatctggccaagct	EMSA
$P_{Unrelated}$-R	cgggtcgatgcggtcccag	
P_{hsaf}- F	attccaaagaatgatccgcgtcg	
P_{hsaf}- R	tggtggtggtcggcccg	

Table S2 All primers used in this study. These primers were used for gene in-frame deletion of *Le*YC36, recombinant protein purification, real-time PCR and EMSA assays.

Sample	peak area	concentration
farnesol standard	3014967	2.5 μM
farnesol standard	11830064	10 μM
farnesol standard	22746370	40 μM
farnesol standard	82432680	160 μM
extracellular extract of *C. krusei*-1	20464327	33.60 μM
extracellular extract of *C. krusei*-2	23134447	39.05 μM
extracellular extract of *C. krusei*-3	27854673	48.68 μM

Table S3 Peak Areas and Corresponding Concentrations of Farnesol Standard and Extracellular Extract of *C. krusei* via HPLC.

Reference

1. Robert X, Gouet P. Deciphering key features in protein structures with the new ENDscript server. *Nucleic Acids Res* 2014;**42**:W320-324. https://doi.org/10.1093/nar/gku316
